# Gene expression profiles compared in environmental and malnutrition enteropathy in Zambian children and adults

**DOI:** 10.1016/j.ebiom.2021.103509

**Published:** 2021-07-29

**Authors:** Paul Kelly, Beatrice Amadi, Kanta Chandwe, Ellen Besa, Kanekwa Zyambo, Mubanga Chama, Phillip I. Tarr, Nurmohammad Shaikh, I Malick Ndao, Chad Storer, Richard Head

**Affiliations:** a Tropical Gastroenterology and Nutrition group, University of Zambia School of Medicine, Nationalist Road, Lusaka, Zambia; b Blizard Institute, Barts & The London School of Medicine, Queen Mary University of London, 4 Newark Street, London, UK; c Departments of Paediatrics, Washington University School of Medicine, St Louis, MO, United States; d Departments of Genetics, Washington University School of Medicine, St Louis, MO, United States

**Keywords:** Environmental enteropathy, Environmental Enteric Dysfunction, malnutrition, RNA sequencing, intestinal transport, intestinal digestion, NADPH oxidase, xenobiotic metabolism

## Abstract

**Background:**

Environmental enteropathy (EE) contributes to growth failure in millions of children worldwide, but its relationship to clinical malnutrition has not been elucidated. We used RNA sequencing to compare duodenal biopsies from adults and children with EE, and from children with severe acute malnutrition (SAM), to define key features of these malnutrition-related enteropathies.

**Methods:**

RNA was extracted and sequenced from biopsies of children with SAM in hospital (*n*=27), children with non-responsive stunting in the community (*n*=30), and adults living in the same community (*n*=37) using an identical sequencing and analysis pipeline. Two biopsies each were profiled and differentially expressed genes (DEGs) were computed from the comparisons of the three groups. DEG lists from these comparisons were then subjected to analysis with CompBio software to assemble a holistic view of the biological landscape and IPA software to interrogate canonical pathways.

**Findings:**

Dysregulation was identified in goblet cell/mucin production and xenobiotic metabolism/detoxification for both cohorts of children, versus adults. Within the SAM cohort, substantially greater induction of immune response and barrier function, including NADPH oxidases was noted, concordant with broadly reduced expression of genes associated with the brush border and intestinal structure/transport/absorption. Interestingly, down regulation of genes associated with the hypothalamic-pituitary-adrenal axis was selectively observed within the cohort of children with stunting.

**Interpretation:**

Gene expression profiles in environmental enteropathy and severe acute malnutrition have similarities, but SAM has several distinct transcriptional features. The intestinal capacity to metabolise drugs and toxins in malnourished children requires further study.

**Funding:**

Bill & Melinda Gates Foundation (OPP1066118)

**Research in context**

**Evidence before this study**

Children with manifestations of malnutrition (i.e., stunting and wasting) may have varying degrees of enteropathy, and adults living in the same disadvantaged environments also have environmental enteropathy. However, the overlap between these enteropathic disorders is unclear and direct comparison of transcriptomic profiles has never been attempted.

**Added value of this study**

We used RNA sequencing analysis of intestinal biopsies from Zambian children with either stunting or wasting to identify pathways that could affect digestion, absorption, barrier function and pathophysiology. While there was much overlap between enteropathy in stunted and wasted children, each enteropathy had specific features: reduced expression of brush border genes related to digestion and absorption in wasted children, and reduced adrenal axis genes in stunted children.

**Implications of all the available evidence**

Childhood malnutrition disorders are accompanied by enteropathies which have specific features. Efforts to improve outcomes from treatment programmes may need to take these into account.

Alt-text: Unlabelled box

## 1 Introduction

Malnutrition in children in low and middle income countries persists on a huge scale. Stunting, defined as impaired linear growth, affects millions of children to the extent that no country in Africa is anticipated to meet the Sustainable Development Goals for childhood stature by 2030 [1]. Wasting, which means loss of fat and lean tissue, is less common, and severe acute malnutrition (SAM) is a medical emergency [2]. Optimal treatment in the community remains associated with mortality rates of at least 4% per episode. After medical complications supervene, in-hospital mortality climbs to 10% or more, and if diarrhoea is superimposed, mortality can exceed 20% per episode [3,4]. Counter-intuitively, malnutrition does not fully respond to nutritional therapy. Stunting rates can only be reduced by 15% at most even with optimally-delivered nutritional care [5] with or without sanitation interventions [6]. It is now widely believed that failure to prevent stunting and inability to reduce mortality in SAM are due to the presence of a highly prevalent underlying enteropathy [7], [8], [9].

Environmental enteropathy (EE, now also often referred to as environmental enteric dysfunction, EED) is an asymptomatic disorder, originally termed ‘tropical enteropathy’ [10,11]. It seems likely that adverse environmental conditions, including frequent exposure to enteropathogens or pathobionts [12], [13], [14], [15], [16], cause intestinal damage, manifest as mucosal inflammation, increased intestinal permeability, microbial translocation and systemic inflammation [15]. Children hospitalised with SAM have high in-hospital and post discharge mortality [4,17], and a severe enteropathy [18] which may include defective glycosylation in the epithelium in the oedematous form of SAM (kwashiorkor) [19]. Recent analysis of gene methylation also implicated changes in glycosylation in kwashiorkor [20]. It is not known if the enteropathy of SAM is a distinct disorder superimposed on the background of EE observed in disadvantaged communities, or if it is pathophysiologically indistinguishable. If these enteropathies are pathophysiologically the same, their transcriptomic profiles should be very similar, and outliers distributed at random. Conversely, if these disorders are distinct, then it should be possible to identify clear differences in pathways and gene sets which characterise each enteropathy, and which might then be amenable to specific therapy. To permit such a comparison it would be important that biopsy collection, RNA extraction, library construction, sequencing and analysis be performed in an identical manner. Here we present a comparative transcriptomic analysis of the small bowel in children with EED and stunting and with SAM, which fulfils those criteria and therefore tests the null hypothesis that there are no meaningful differences in mRNA content between these enteropathies. As we had no ethical justification to collect biopsies from healthy children, these two groups are compared with adults from the same community as the stunted children.

## 2 Methods

We directly compared environmental enteropathy in three groups: hospitalised children with SAM and persistent diarrhoea, asymptomatic children with stunting unresponsive to nutritional therapy, and apparently healthy adults from a community in which EE is known to be almost ubiquitous. The last two groups were recruited exclusively from the same disadvantaged community (Misisi, Lusaka), and the groups have been described in previous publications [16,21,22]. The adults and children from Misisi were in apparently good health; potential participants with clinical illness did not donate samples for this study. The adults were volunteers [21], and the children had been requested to undergo endoscopy and biopsy in order to search for infections or other explanations for severe acute, or chronic non-responsive, malnutrition [16]. The presence of EE in each group was confirmed using formal morphometry of mucosal biopsies.

### 2.1 Ethics

Ethics approval was obtained from the University of Zambia Biomedical Research Ethics Committee on 11^th^ April 2013, reference number 006-01-13, and 31^st^ May, 2016, reference 006-02-16. Written, informed consent was obtained from participants, parents or caregivers in all cases.

### 2.2 Study participants

SAM was defined as weight-for-length *z* score < -3, mid upper arm circumference <115 mm, or bilateral pedal oedema. Stunting was defined as length-for-age *z* score <-2. Children with SAM and persistent diarrhoea were recruited from the malnutrition ward of the University Teaching Hospital, Lusaka, as previously described [22]. This ward treats children with SAM accompanied by medical complications; uncomplicated SAM is treated in the community. Consequently, these children have severe disease and mortality rates have historically been high. Up to one third of the children on this ward have HIV infection [18], further increasing mortality [4,17]. Children with stunting were recruited from Misisi, a residential area of Lusaka, Zambia, in which we have conducted previous studies of environmental enteropathy [21]. They were investigated for non-response to nutritional interventions over 4-6 months [16]. In all cases initial investigations (including stool microscopy for protozoa) were negative, so endoscopy (with biopsy) was performed to identify treatable enteropathogens and hypolactasia (lactase deficiency). Adults were also recruited from Misisi and were asymptomatic at the time of endoscopy. Recruitment of adults and consent processes included community-based group discussions [23]. Nutritional assessment in adults included height, weight, and MUAC, and from these BMI was calculated and stunting defined as length-for-age *z* score <-2 using WHO height standards for 19-year olds (https://www.who.int/tools/growth-reference-data-for-5to19-years, accessed 14^th^ April 2021).

### 2.3 Investigations in children with stunting

Children in Misisi between 0 and 18 months of age were screened for early malnutrition, anthropometry performed (using weight, height and mid upper arm circumference, MUAC) and recruited if they had WAZ, LAZ and/or WLZ scores below -2 (i.e., 2 standard deviations below the mean for age using WHO growth charts). These children were included in a nutritional rehabilitation programme which provided corn-soy blend, a micronutrient sprinkle, and a daily egg. Children whose LAZ or WLZ scores remained consistently <-2 over 4-6 months were invited for endoscopy to search for treatable causes of the refractory malnutrition [16]. Endoscopy was performed under ketamine-based sedation, administered by an anaesthetist, with a Pentax EG2490k gastroscope (external diameter 8mm) [24]. Biopsies were collected from the distal duodenum.

### 2.4 Investigations in children with SAM

Children with SAM and persistent diarrhoea, but with stool tests that failed to show a treatable pathogen, whose parents or guardians had given written consent underwent endoscopy [22]. Endoscopy and anthropometry were performed as for the children with stunting.

### 2.5 Investigations in adults

In consenting adults, a thorough clinical assessment was carried out, including anthropometry (weight, height, MUAC). HIV serological testing was performed with consent, following national guidelines. Exclusion criteria included concurrent illness, pregnancy, use of antibiotics or nonsteroidal anti-inflammatory drugs within one month before the date of endoscopy, or recent helminth infection. Endoscopy was performed under conscious sedation using a Pentax EG2990i gastroscope and biopsies were collected from the distal duodenum [21].

### 2.6 RNA sequencing

A consistent protocol for biopsy handling was used for all RNA sequencing. Two duodenal biopsies were immediately snap-frozen in liquid nitrogen in the endoscopy unit, and stored at -80°C until RNA extraction; extraction was performed using Trizol reagent (Invitrogen, Carlsbad, CA) followed by silica column purification (RNeasy Mini Kit, Qiagen), and quantified (Nanodrop spectrophotometer, ND-1000, Thermo Scientific, Minneapolis, MN), before transport to the Beijing Genomics Institute (BGI) on dry ice. RNA quality control was performed using an Agilent 2100 Bioanalyser and ABI StepOnePlus Real-Time PCR System. For RNA sequencing preparation, total RNA was treated with DNase I followed by mRNA enrichment using oligo-dT labelled beads and ligation of sequencing adaptors to the enriched mRNA fragments. RNA sequencing was performed using an Illumina HiSeq2000 platform. Filtering steps included removing reads with adapters, removing reads in which unknown bases were more than 10%, and removing low quality reads (when low quality bases were over 50%). The proportion of clean reads was ≥ 99%, and usually >99.6%. After removing low-quality reads and trimming adapter sequences, 3.3 billion clean reads were obtained, corresponding to an average of 165 million reads per sample.

### 2.7 Statistics

Expression levels for each gene were expressed as Fragments Per Kilobase Mapped (FPKM). FPKM data were normalized across samples using the quantile normalization functionality in the Partek Genomics Suite (Partek, www.partek.com). Gene expression profiles were compared between adults and children, and between children with acute malnutrition (SAM) or non-responsive stunting. To do this, we calculated the mean FPKM value for each gene in each of the three groups, calculated the three ratios between each pair of groups, and identified transcripts with FPKM values outside 95% confidence limits of the ratio. We trimmed lists of genes with ratios outside this range by excluding genes for which one of the FPKM values were less than 1. In a second analytical approach, we also identified those transcripts with an absolute fold-change greater than 2.5-fold and a nonparametric p-value significance less than 1E-6 (Mann-Whitney, adjusted p-value less than 0.02) as an intentionally strict cut-off intended to identify only the most salient differences between the groups. We used CompBio V2.0 (PercayAI, https://www.percayai.com/, accessed on 8^th^ February 2021) [25], [26], [27] to identify biological themes in each gene set of differentially expressed genes (as identified using -fold change) to identify knowledge/context-based concepts and themes present between, and unique to, the pairwise comparisons of the groups. Ingenuity Pathway Analysis (QIAGEN, https://www.qiagenbioinformatics.com) was also used to compare the three groups. No sample size calculation was carried out. Comparison of HIV positive and negative transcriptomes was carried out using NOIseq. All authors had access to study data and approved the final submission.

### 2.8 Role of the funding source

This study was supported by the Bill & Melinda Gates Foundation (OPP1066118), which played no role in the analysis of results or the decision to publish.

## 3 Results

**Fig. 1:**
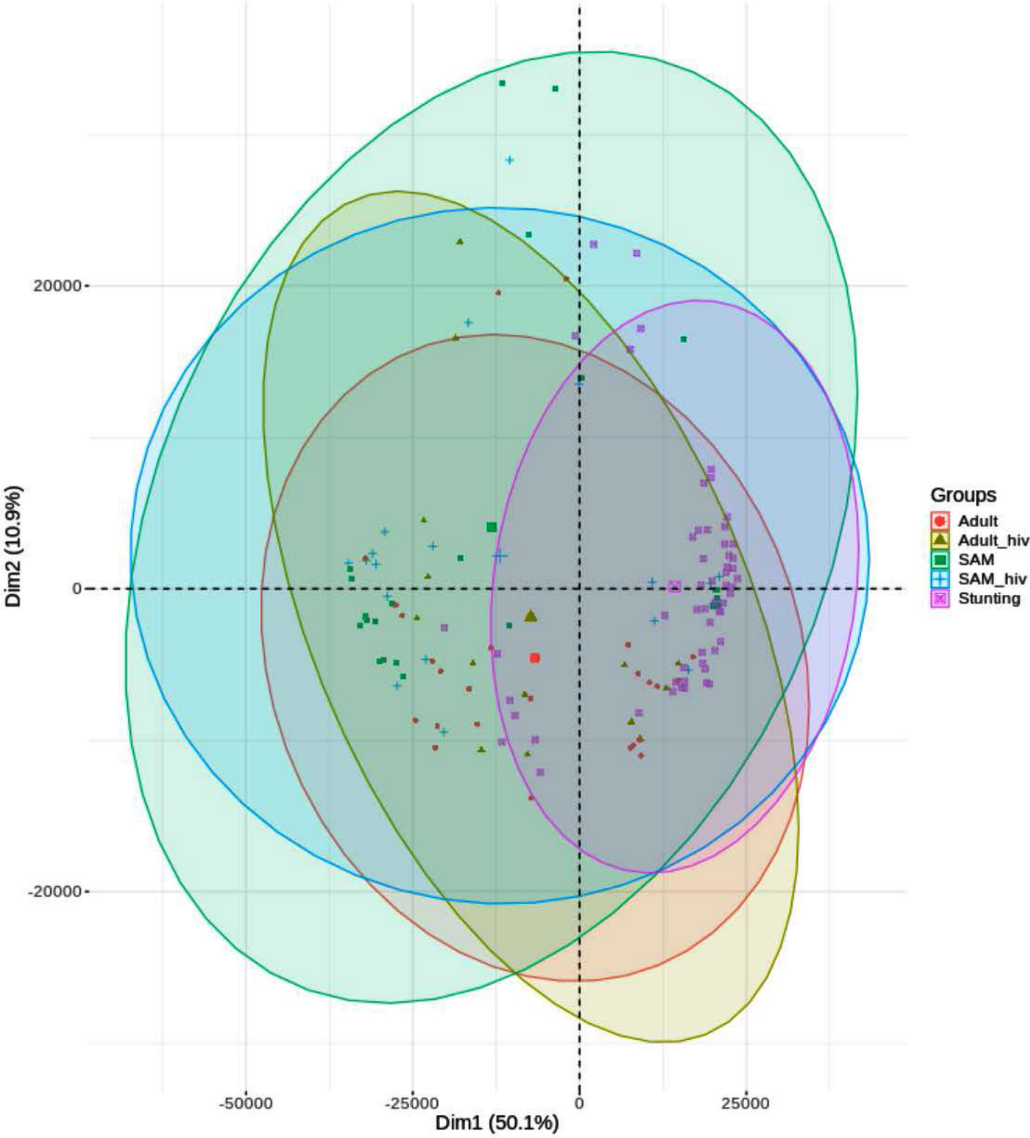
Principal Components Analysis (PCA) of annotated gene expression levels, with a median expression >= 1.0 in at least one cohort. Ellipses represent 95% confidence interval for the indicated cohort. Note that there were no recorded HIV-positive individuals in the stunting group.

Transcriptomic profiling was performed on biopsy sets from 30 children with non-responsive stunting, 27 hospitalised children with SAM and persistent diarrhoea, and 38 adults from the same community as the stunted children with stunting (Table 1). Children with SAM and those with stunting were similar in age (Table 1). A total of 20,120 transcripts were mapped to the reference genome. Only 16 genes were differentially expressed by HIV status in adults (Table S1), and PCA analysis demonstrated a high degree of overlap between HIV infected and uninfected adults and SAM children (Fig. 1), so HIV status was not considered in further analysis.

**Table 1:** Nutritional status and laboratory investigations of children with undernutrition and adults from the same community as the children with stunting

	Children with stunting	Children with SAM	Adults
n	30	27	37
Sex (M:F)	15:15	16:11	11:26
Age	13 (5,15) months	15 (10,21) months	28 (23,43) years
HIV infected (n,%)	0	9 (33%)	12 (32%)
BMI (kg/m^2^)	-	-	22.8 (21.7,24.9)
WAZ	-2.2 (-2.7,-1.7)	-3.4 (-5.0,-2.7)	-
LAZ	-3.3 (-3.9, -2.8)	-2.8 (-3.9,-1.7)	-
WLZ	-0.62 (-1.3,-0.1)	-2.8 (-4.2, -1.6)	-
MUAC (cm)		10.8 (10.3, 12.3)	26.8 (25.6,28.6)
Oedema present (n, %)	0	12 (44%)	0
Height (m)			Female: 1.57 (1.54,1.64) Male: 1.65 (1.60,1.70)
Stunted (n, %)	30 (100%)	19 (70%)	Female: 1 (4%) Male: 6 (55%)
Villus height (VH, μm)	186 (135, 199) [range 89-220]	215 (164, 246) [range 74-284]	215 (200, 259) [range 151-310]
Crypt depth (CD, μm)	169 (148, 205) [range 115-234]	163 (144, 195) [range 106-220]	167 (142, 182) [range 125-218]
VH:CD ratio	1.02 (0.86, 1.28) [range 0.48-1.58]	1.33 (0.96, 1.53) [range 0.41-1.89]	1.38 (1.22, 1.50) [range 0.89-2.25]
Taking HIV anti-retroviral therapy prior to date of biopsy	-	3 (33%)	5 (42%)

All continuous variables are given as median and interquartile range (IQR). Significance testing was not performed as differences between groups were a consequence of recruitment criteria. Mucosal morphometry was available on a subset of well-orientated sections (n=24 in children with stunting, n=18 in children with SAM, and n=29 in adults). For reference ranges of mucosal morphometry, see reference [28] which suggests approximate normal values of 264-424μm for VH, and 120-220μm for CD in British children.

### 3.1 Comparison of gene expression in children with stunting and adults from the same community

**Fig. 2:**
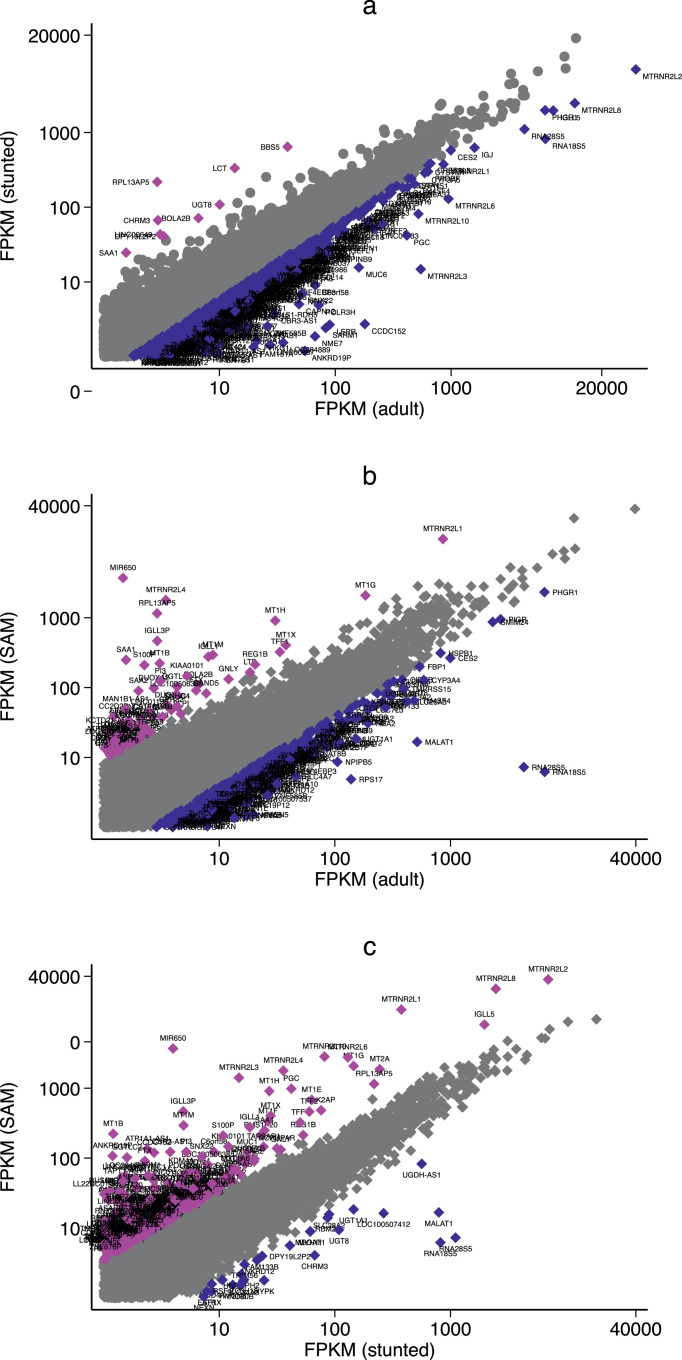
Scatter plots of paired expression comparisons between (a) Children with stunting vs. adults from the same community (ρ = 0.96; *P*<0.0001), (b) Children with SAM vs adults (ρ = 0.94; *P*<0.0001), (c) Children with SAM vs. stunting (ρ = 0.94; *P*<0.0001). Genes shown are classified as those with ratios within 95% confidence limits for ratio (grey) and those outside the 95% confidence limit of the ratio (blue or magenta). Gene symbols denote genes with ratios falling outside 95% confidence limits. Only genes with FPKM >1 for both groups are shown; Tables S2-7 present the full lists of differentially expressed genes in which only one has FPKM>1 .

**Fig. 3:**
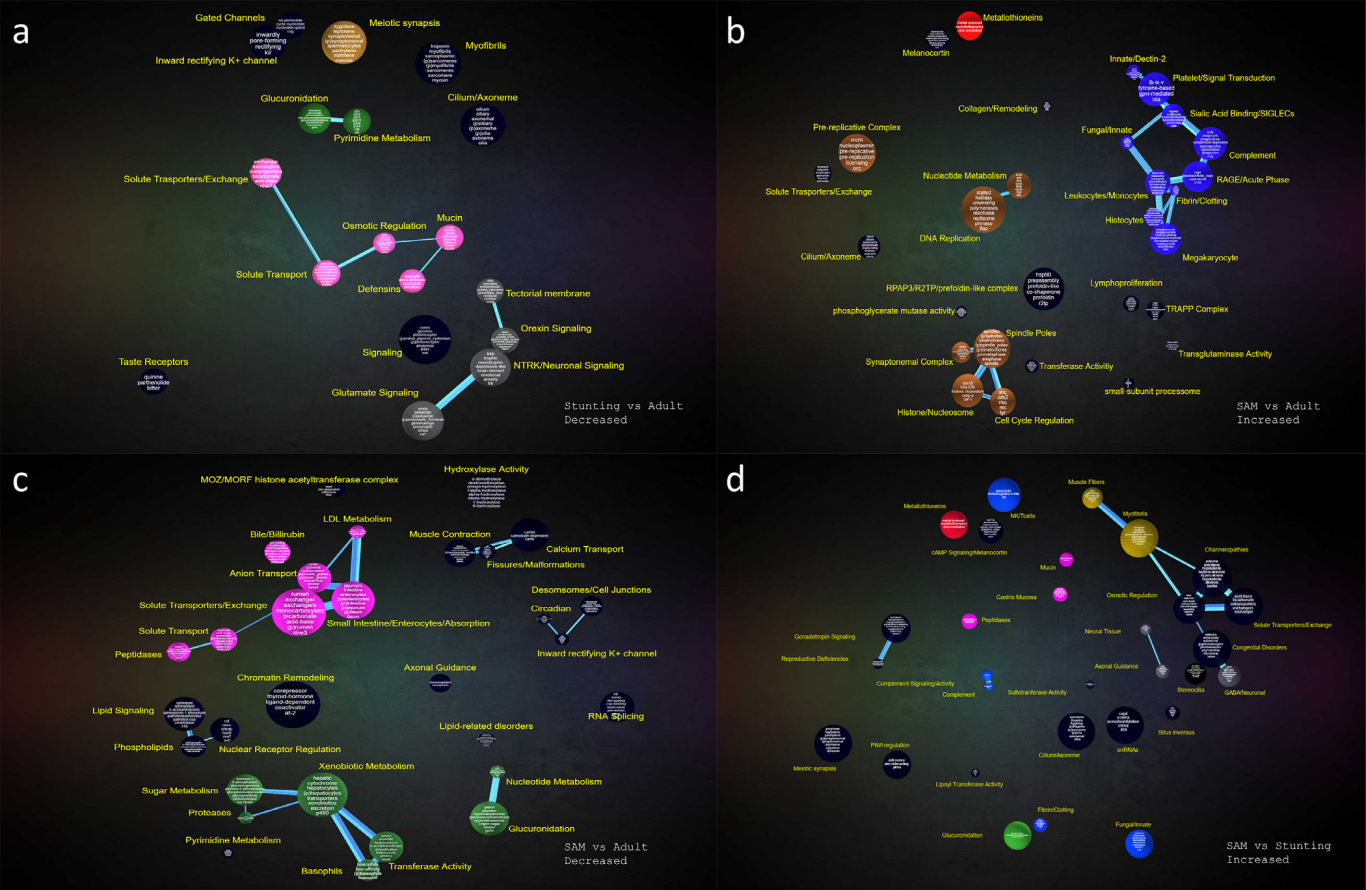
CompBio functional analysis of gene expression in selected pairwise comparisons between groups; pairwise comparisons with limited numbers of differentially expressed genes are not shown. (a), genes with reduced expression in children with stunting compared to adults from the same community. (b), genes with increased expression in children with SAM compared to adults from the same community. (c), genes with reduced expression in children with SAM compared to adults from the same community. (d), genes with increased expression in children with SAM compared to children with stunting. Related interconnected themes were assigned to groups as follows: gastric/mucus (pink), immune (blue), xenobiotic metabolism (green), metallothionein (red), DNA replication(brown), enteric nervous system (grey), muscle fibers (gold).

In the first analysis, transcriptomes of asymptomatic children with stunting were compared to adults from the same community. FPKM values were highly correlated so we derived the ratio (children with stunting:adults) of FPKM values for each transcript, and then identified those that fell outside the 95% confidence limits of the ratio (Fig. 2a). We identified 501 genes with increased expression in children with stunting compared to adults, of which 58 had either FPKM value greater than 1 (Table S2). Likewise, we identified 503 genes with reduced expression in children with stunting compared to adults; of these 326 had FPKM values in either group >1 (Table S3). Lactase (LCT) was predictably expressed at much greater relative values in children than in adults (Fig. 2a), though it only met the 95% confidence threshold in the stunted group. There was also greater expression of 6 olfactory receptor genes, two solute carriers (a nucleotide-sugar cotransporter and an organic anion transporter), galanin receptor 3, a gap junction protein and one homeobox gene (Table S2). Genes expressed at a lower values in children with stunting included genes for the polymeric immunoglobulin receptor (PIGR), a broad range of xenobiotic metabolising enzymes, steroid metabolism, mucus biology and metallothioneins (Table S3, Fig. 3a). Interestingly, transcripts for a range of hormone receptors (receptors for leptin, vasoactive intestinal polypeptide, secretin and gonadotrophin releasing hormone) were also reduced (Table S3), and SMADs (required for Transforming Growth Factor β signalling) were reduced in children with stunting.

### 3.2 Comparison of gene expression in children with SAM and adults

We then compared gene expression between children with SAM and adults (Fig. 2b). We identified 503 genes with increased expression in SAM enteropathy, of which 222 remained after trimming (Table S4), and 503 genes with reduced expression in SAM enteropathy compared to adults, of which 180 survived trimming (Table S5). Genes in SAM that were relatively over-expressed include NADPH oxidases, dendritic cell-specific loci, chemokines, metallothioneins, trefoil factors, metalloproteases, and membrane ion transport (Fig. 3b and Table S4). CompBio analysis demonstrated reduced expression of large sets of genes responsible for xenobiotic metabolism and membrane transporters (Fig. 3c and Table S5).

### 3.3 Comparison of gene expression in children with SAM and children with stunting

To identify the effect of SAM complicated by diarrhoea over and above the environmental enteropathy present in stunting we compared children with SAM and children with stunting only (Fig. 2c). We identified 503 genes with increased expression in SAM enteropathy compared to children with stunting, and 502 genes with reduced expression in SAM enteropathy; after trimming there remained 385 increased in SAM (Table S6) and 80 transcripts reduced in SAM (Table S7). Genes more highly expressed in SAM grouped into antimicrobial defence, mucosal protection, chemokines, metallothioneins, and NADPH oxidase function (Fig. 3d, Table S6). Genes with reduced expression in SAM included three UDP glycosyltransferases, seven olfactory receptors, galanin receptor 3, aquaporin 12B, and a broad range of ribosomal RNA genes (Table S7).

### 3.4 Comparison of specific domains of intestinal function: brush border digestive enzymes, solute carriers and xenobiotic metabolising enzymes

**Fig. 4:**
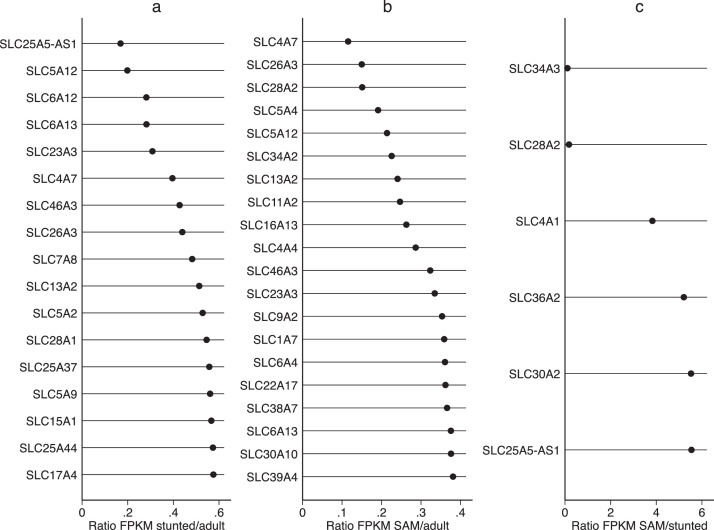
Dot plots of ratios of FPKM values for solute carrier (SLC) genes identified in intestinal biopsies from (a) children with stunting compared to adults from the same community, (b) children with SAM compared to adults, and (c) children with SAM compared to children with stunting. Genes shown are only for genes with ratio falling outside 95% confidence limits and where both genes are expressed at FPKM >1. Note that in panels a and b, no SLC genes had a ratio >1 under the conditions stated.

**Fig. 5:**
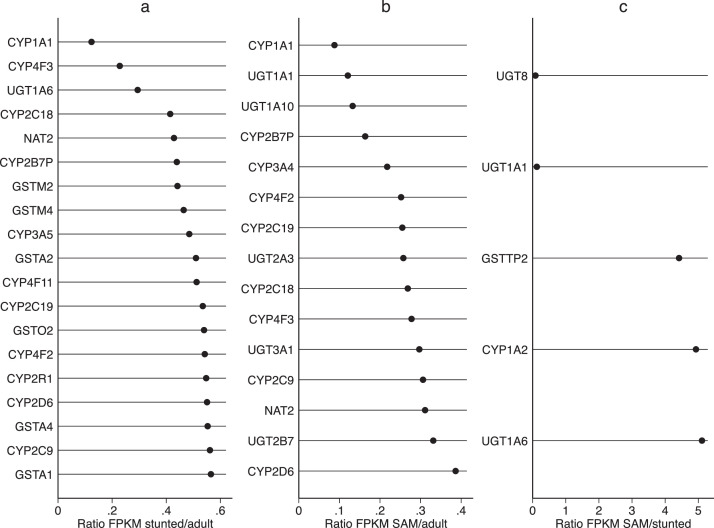
Dot plots of FPKM values for xenobiotic metabolism genes identified in intestinal biopsies from (a) children with stunting compared to adults from the same community, (b) children with SAM compared to adults, and (c) children with SAM compared to children with stunting. Genes shown are only for genes with ratio falling outside 95% confidence limits and where both genes are expressed at FPKM >1. Note that in panels a and b, no xenobiotic metabolism genes had a ratio >1 under the conditions stated.

As we have previously reported correlations between severity of enteropathy in SAM and specific domains of enteropathy [22], we compared expression of solute carriers (SLCs), digestive enzymes, and xenobiotic metabolising enzymes across the three groups. Many SLCs were reduced in both childhood groups compared to adults (Fig. 4) but transporters for glycine/proline, bicarbonate, and zinc were increased in children with SAM compared to stunting. A similar pattern was observed for xenobiotic metabolising enzymes (Fig. 5). In an exploratory analysis of known brush border enzymes these were broadly reduced in SAM (Table 2), although only six (LIPF, DPP4, MEP1B, SI, ACE and TMPRSS15) lay outside 95% confidence limits of the ratio with adults.

**Table 2:** Digestive enzyme genes in the three groups

Location of usual expression	Name	Enzyme	Gene	Mean FPKM by group		
				Stunting	SAM	Adult
Pancreatic proteases	Cationic trypsinogen	3.4.21.4	PRSS1	0.2	9.8	0.1
	Anionic trypsinogen	3.4.21.4	PRSS2	267.9	168.6	87.2
	Mesotrypsinogen	3.4.21.4	PRSS3	133.6	158.1	185.4
	Pepsinogen C	3.4.23.1	PGC	40.6	509.7	330.7
	Pancreatic procarboxypeptidase A2	3.4.17.15	CPA2	20.7	20.8	16.8
	Pancreatic procarboxypeptidase A3	3.4.17.1	CPA3	1.6	2.1	2.3
Brush border proteases	Enteropeptidase	3.4.21.9	TMPRSS15	261.2	91.4	459.6
	Dipeptidylpeptidase IV	3.4.14.5	DPP4	67.7	31.3	88.3
	γ-glutamyl transferase	2.3.2.2	GGT1	172.2	86.8	128.7
	Aminopeptidase A	3.4.11.7	ENPEP	49.6	20.3	47.4
	Aminopeptidase N (alanyl amino-peptidase)	3.4.11.2	ANPEP	2391.7	1504.7	3321.1
	Glutamate carboxypeptidase II (GP2), folate hydrolase	3.4.17.21	FOLH1	201.6	134.6	206.1
	Angiotensin 1 converting enzyme	3.2.1 / 3.4.15.1	ACE	180.0	107.0	314.5
	Neprilysin, membrane metalloendopeptidase	3.4.24.11	MME	135.4	62.4	155.6
	Meprin A	3.4.24.63	MEP1B	146.4	52.3	220.9
Brush border enzymes not protease	Alkaline phosphatase, intestinal	3.1.3.1	ALPI	1382.1	555.0	1123.0
	Sucrase-isomaltase	3.2.1.20	SI	163.4	50.6	307.2
	Maltase-glucoamylase	3.2.1.20	MGAM	101.6	36.7	102.9
	Lactase	3.2.1	LCT	363.1	87.7	14.0
	Trehalase	3.2.1.28	TREH	177.5	101.9	270.1
Lipases	Gastric lipase	3.1.1.3	LIPF	0.2	1.5	7.2

Genes with FPKM values <1.0 are omitted. Only 4 genes (LIPF, CPA2, PGC, and PRSS2) did not achieve signficance ≤ 0.05 in a non-parametric test for significance of the ratio SAM: adults.

### 3.5 Summary of pathway analysis

**Fig. 6:**
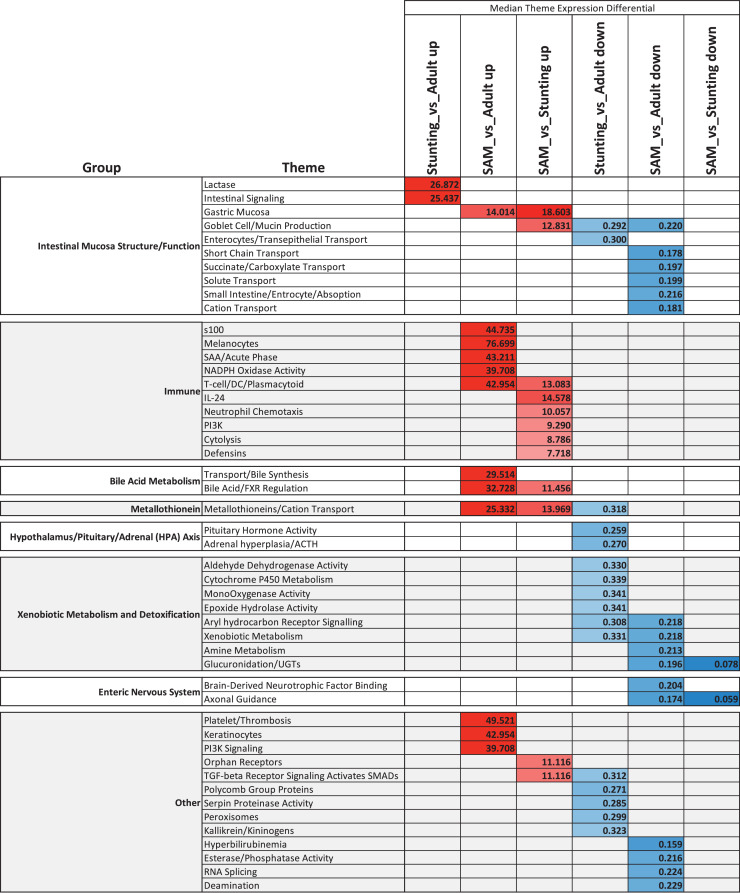
Heatmap summarising the results of the CompBio analysis across the three cohort comparisons. The value within each cell is the median differential expression for all genes associated with the given enriched theme for that cohort comparison; red shading represents increased and blue shading represents decreased. CompBio themes were further grouped into over-arching biological processes.

A summary of the CompBio analysis (Fig. 6) demonstrates many common pathophysiological features between the enteropathies of acute and chronic malnutrition in children, but unique features of SAM included upregulation of FXR/bile acid metabolism, increased expression of metallothioneins, a broadly up-regulated immune response profile and a down-regulated enterocyte/transport profile. Conversely, the EED/stunting children exhibited a reduced expression of genes involved in the hypothalamic-pituitary-adrenal axis (HPA), most notably luteinizing hormone B (*LHβ*).

We also performed a core analysis of the three enteropathies using Ingenuity Pathway Analysis software on lists of identified differentially expressed genes (Fig. S1). In this analysis xenobiotic metabolism and FXR/LXR/RXR nuclear receptor signaling were the primary signals. Xenobiotic metabolism was broadly significantly decreased in the stunting and SAM groups compared with adults, and further decreased in the SAM group over the stunted group.

## 4 Discussion

Environmental enteropathy and the enteropathy observed in children with severe acute malnutrition belong to a spectrum of enteropathies characterised by villus blunting and fusion, lymphocytic infiltration, and epithelial disruption [18,21,[28], [29]. Functionally, these enteropathies are characterised by increased permeability [30], [31], [32], microbial translocation [33], [34], and impaired nutrient uptake [35], [36]. Many different forms of enteropathy are now described, including immunologically-mediated (coeliac disease, autoimmune, graft-versus-host disease, transplantation rejection enteropathy), infective enteropathies, drug-induced enteropathy (checkpoint inhibitor enteropathy), and those due to disturbed prostaglandin action (SLCO2A1 and non-steroidal anti-inflammatory drug enteropathies). Superficially these are histologically similar, so a molecular approach to their classification would be highly desirable. If the environmental enteropathy observed in children with stunting, and the enteropathy observed in children with SAM were essentially nosologically identical, any transcriptional differences between them would be minor and randomly distributed throughout the transcriptome. In contrast, we found many common features, but also specific signatures which characterise the enteropathy of SAM. These include the increase in NADPH oxidase transcription and the increased expression of mucus-related genes, which may reflect increased demand. Interestingly, these are also features of Crohn's disease [37], [38]. In paediatric ileal Crohn's disease the dominant signature was represented by *DUOX2*, which associated with increased inflammatory genes, alongside reduced lactase and cytochrome P450 genes [37]. Several DEGs, including *SAA1* and *SAA4, MUC1, DUOXA1, CEACAM7* and *S100A8*, are over-represented in both children with SAM and children with Crohn's. Upregulation of *MUC1* and *MUC4* was also identified in uninvolved colon in IBD [38], and changes in glycosylation of MUC1 have been implicated in IBD-associated neoplasia [39]. The dramatically increased expression of gastrokines is as yet of uncertain physiological significance. However, solute carriers, the major nutrient transporters that drive intestinal absorption are similarly expressed between the groups of children. There appears to be a broad and consistent reduction in brush border digestive enzymes in SAM. If these differences in mRNA can also be demonstrated at the level of protein expression, then we might infer that SAM enteropathy has additional pathophysiological characteristics and may require different interventions. This enteropathy could therefore be termed malnutrition enteropathy, but this does not imply that the malnutrition causes the enteropathy, for which additional evidence would be required. Future work should study children with SAM and without diarrhoea as the pathogens driving persistent diarrhoea probably also complicate interpretation of these transcriptomic data.

While expression of luteinizing hormone beta (LHβ) is generally thought of as a product of the pituitary gland, clear expression was observed in these biopsies, and was significantly reduced in the SAM cohort as compared with the adult group. CYP21A2 was also observed to be down regulated in stunted children as compared with adult EED. CYP21A2 is involved in, among other things, steroid hydroxylation. Loss of CYP21A2 21-hydroxylase activity is known to affect adrenal gland hormone production and growth. While the relevance of these observations is not currently understood, the role of LHβ and CYP21A2 in the HPA-axis is clear, as is the role of the HPA-axis in growth. Two other transcriptomic analysis of children with stunting in Pakistan [40] and Malawi [41] provide substantial corroboration for the observed patterns of gene expression reported here. In Pakistan [40], children with stunting were compared to American children. Reduced expression of xenobiotic and antioxidant genes was observed, together with increased expression of antimicrobial proteins (DUOX family), some MUC genes, and interferons. Digestive enzymes (LIPF and SI) were reduced in Pakistani children with stunting, similar to our findings (Table 2). Differential expression of xenobiotic, antioxidant and DUOX genes clearly discriminated EE from celiac disease. In Malawian children with stunting, transcriptomic analysis of stool identified over-expression of mucin and inflammatory genes [41]. There seems to be an emerging understanding that mucin biology is key to understanding mucosal inflammation in several disorders, including EE.

Children with stunting and children with SAM have common transcriptomic features when compared to the adult cohort, such as reduced solute carrier and xenobiotic enzyme gene expression. We have previously reported that solute carrier mRNA is increased in the most severe forms of enteropathy in children with SAM [22], but in the inter-group comparison reported here a substantial number of SLCs were reduced in both groups of children with malnutrition. Without healthy child controls we cannot confidently determine if changes observed in both groups of children are caused by pathology or to the immaturity of the intestine. There are marked histopathological abnormalities in children with stunting and enteropathy [18,29] but we cannot exclude the possibility that age-related changes in transcription may be reflected in these data, just as observed for lactase expression.

Metallothionein genes demonstrated remarkably robust increased gene expression in the biopsies of SAM children as compared with the adult cohort. Taken together with changes in several zinc transporter families (SLC11A1, SLC30 family and SLC39 family) these data underscore the singular importance of zinc to intestinal health and function. Zinc is critical for Paneth cell granule integrity, and in malnourished children. Zinc absorption is reduced in children with stunting [35], and has demonstrable clinical benefits in children with diarrhoea [42]. It should be noted that in the periods leading up to biopsy collection as part of this study, both groups of children received nutritional supplements, including zinc. Whether this might explain the differential expression of metallothioneins is not clear, as metallothionein expression in blood cells can be modulated by zinc exposure in vivo and in vitro [43]. Further work is required on the impact of zinc on intestinal expression of genes involved in zinc absorption.

The ability to metabolise toxins and drugs is also important for children living in insanitary environments. As no biopsies were available from completely healthy children living in clean surroundings, we cannot comment on the normal expression values of xenobiotic enzymes in children, and therefore cannot claim that expression is impaired. However, the need to investigate this further is obvious. Altered metabolism of xenobiotics may lead to reduced ability to detoxify environmental toxins, including widely prevalent food-borne aflatoxins and chemical pollutants. Over the long term this could increase predisposition to non-communicable disease, including gastrointestinal and liver cancers. Of immediate consequence, such changes may lead to changes in the pharmacokinetics of orally-administered drugs.

In addition to the described limitations of the comparator cohorts, it should also be noted that the differentially expressed gene (DEG) selection criteria were intentionally stringent to yield gene lists of tractable sizes, focusing on the most salient differences between the three cohorts. With the use of thresholds for selection, it is possible and likely that DEGs meeting the criteria for one cohort comparison may miss the threshold for another, even if the DEG is considered significant by statistical evaluation. Thus, apparent absence of a DEG may be due to lack of a differential within that cohort, or simply failing to meet the threshold criteria. Finally, while CompBio is a valuable tool for biological process analysis [25], [26], [27], because it harvests information on pathways and linkages through comprehensive interrogation of all published biological knowledge, no tool for pathway analysis can analyse genes for which little information on function is available, including enigmatic genes such as gastrokines which are dramatically overexpressed in SAM. Bulk transcriptomic analysis, such as reported here, also tends to average out expression signals from less abundant cell types such as secretory cells (goblet cells and Paneth cells) and less common lymphocyte subsets. Nevertheless, these data do provide helpful insights into the commonalities and differences between different forms of enteropathy seen in very vulnerable children.

## Declaration of Competing Interest

PIT is a consultant to, and holder of equity in MediBeacon Inc, which is developing novel technology to measure intestinal permeability in humans under a patent he partly owns. RDH and CS may receive royalty income based on the CompBio technology they developed, which was licensed by Washington University to PercayAI. All other authors declare no competing interests.

## Appendix Supplementary materials

Image, application 1
