# Supplementary material for: Gene expression profiles compared in environmental and malnutrition enteropathy in Zambian children and adults
Source: eBioMedicine. 2021 Jul 29;70:103509. doi: 10.1016/j.ebiom.2021.103509 (PMC8346547; doi:10.1016/j.ebiom.2021.103509)
Supplement: Supplementary file 1 [file mmc1.docx]

**Molecular classification of enteropathy in Zambian children with malnutrition**

**Supplementary material**

Table S1 Differentially expressed genes by HIV infection in adults

Table S2 Genes with increased expression in stunted children compared to adults with EE

Table S3 Genes with reduced expression in stunted children compared to adults with EE

Table S4 Genes with increased expression in children with SAM compared to adults with EE

Table S5 Genes with reduced expression in children with SAM compared to adults with EE

Table S6 Genes with increased expression in children with SAM compared to children with stunting

Table S7 Genes with reduced expression in children with SAM compared to children with stunting

Figure S1 Ingenuity Pathway Analysis showing consistency of many of the transcriptional themes with Compbio.

**Table S1 Differential gene expression by HIV status**

| Mean FPKM HIV positive | Mean FPKM HIV negative | log2ratio (HIVpos/neg) | Probability | Symbol | Description |
| --- | --- | --- | --- | --- | --- |
| 23.16444444 | 1.796153846 | 3.688929251 | 0.840 | EIF3CL | eukaryotic translation initiation factor 3, subunit C-like |
| 34.15888889 | 4.050769231 | 3.075993245 | 0.831 | IFI44L | interferon-induced protein 44-like |
| 980.8177778 | 140.3607692 | 2.804845369 | 0.856 | IFI6 | interferon, alpha-inducible protein 6 |
| 40.68111111 | 6.375384615 | 2.673774799 | 0.815 | REG1B | regenerating islet-derived 1 beta |
| 39.22111111 | 6.296923077 | 2.638911458 | 0.813 | IFI44 | interferon-induced protein 44 |
| 570.1522222 | 103.0046154 | 2.468638166 | 0.836 | HBA2 | hemoglobin, alpha 2 |
| 588.0322222 | 122.9930769 | 2.257318101 | 0.819 | ISG15 | ISG15 ubiquitin-like modifier |
| 15.70111111 | 97.61153846 | -2.636185039 | 0.835 | HLA-DRB4 | major histocompatibility complex, class II, DR beta 4 |
| 0.942222222 | 13.10076923 | -3.797440356 | 0.804 | CR2 | complement component (3d/Epstein Barr virus) receptor 2 |
| 1.066666667 | 15.62384615 | -3.872568339 | 0.822 | CD22 | CD22 molecule |
| 1.885555556 | 28.63384615 | -3.924659906 | 0.862 | VPREB3 | pre-B lymphocyte 3 |
| 1.007777778 | 16.79153846 | -4.058484963 | 0.834 | LIPF | lipase, gastric |
| 2.577777778 | 99.46923077 | -5.270050516 | 0.936 | FDCSP | follicular dendritic cell secreted protein |
| 0.984444444 | 43.23230769 | -5.456656245 | 0.922 | CXCL13 | chemokine (C-X-C motif) ligand 13 |
| 0.246666667 | 19.15230769 | -6.278811655 | 0.901 | MS4A1 | membrane-spanning 4-domains, subfamily A, member 1 |
| 0.076666667 | 8.966923077 | -6.869869756 | 0.846 | TCL1A | T-cell leukemia/lymphoma 1A |

**Table S2 DEGs increased in children with stunting compared to adults**

| genename | description | meanfpkm_stunted | meanfpkm_adult | ratiostuntedadult |
| --- | --- | --- | --- | --- |
| LCT | lactase | 333.4492 | 13.5726 | 24.5679 |
| RPL13AP5 | ribosomal protein L13a pseudogene 5 | 218.2280 | 2.9041 | 75.1447 |
| MRGPRX3 | MAS-related GPR, member X3 | 22.1492 | 0.8964 | 24.7087 |
| CECR7 | cat eye syndrome chromosome region, candidate 7 (non-protein coding) | 4.2315 | 0.1497 | 28.2585 |
| LOC644172 | mitogen-activated protein kinase 8 interacting protein 1 pseudogene | 10.2827 | 0.1428 | 71.9974 |
| VENTXP7 | VENT homeobox pseudogene 7 | 5.8329 | 0.1364 | 42.7598 |
| LINC00639 | long intergenic non-protein coding RNA 639 | 3.3866 | 0.1328 | 25.4976 |
| SLC35G3 | solute carrier family 35, member G3 | 5.5851 | 0.0892 | 62.5915 |
| SLCO1A2 | solute carrier organic anion transporter family, member 1A2 | 1.8039 | 0.0772 | 23.3728 |
| MYADML | myeloid-associated differentiation marker-like (pseudogene) | 2.1063 | 0.0713 | 29.5484 |
| ZFR2 | zinc finger RNA binding protein 2 | 2.1673 | 0.0697 | 31.0751 |
| ANKRD30BL | ankyrin repeat domain 30B-like | 1.9947 | 0.0644 | 30.9941 |
| CCT8L2 | chaperonin containing TCP1, subunit 8 (theta)-like 2 | 2.2102 | 0.0641 | 34.4786 |
| VPREB1 | pre-B lymphocyte 1 | 1.5956 | 0.0641 | 24.8913 |
| LINC01191 | uncharacterized LOC440900 | 3.4242 | 0.0564 | 60.7024 |
| ACTR3BP2 | ARP3 actin-related protein 3 homolog B (yeast) pseudogene 2 | 4.8334 | 0.0513 | 94.2511 |
| ZNF541 | zinc finger protein 541 | 1.3681 | 0.0487 | 28.0828 |
| LOC440311 | glioma tumor suppressor candidate region gene 2 pseudogene | 2.8932 | 0.0477 | 60.6643 |
| HIST2H3D | histone cluster 2, H3d | 1.0976 | 0.0444 | 24.7442 |
| HUS1B | HUS1 checkpoint homolog b (S. pombe) | 1.2622 | 0.0438 | 28.7871 |
| PCDHA5 | protocadherin alpha 5 | 1.1115 | 0.0418 | 26.5948 |
| DMBX1 | diencephalon/mesencephalon homeobox 1 | 1.2085 | 0.0415 | 29.0929 |
| RNF5P1 | ring finger protein 5, E3 ubiquitin protein ligase pseudogene 1 | 1.3002 | 0.0403 | 32.2972 |
| MT3 | metallothionein 3 | 1.1339 | 0.0341 | 33.2496 |
| FAM138B | family with sequence similarity 138, member B | 1.1547 | 0.0328 | 35.1837 |
| WWC2-AS2 | WWC2 antisense RNA 2 | 1.2920 | 0.0323 | 39.9915 |
| PRSS38 | protease, serine, 38 | 1.0798 | 0.0323 | 33.4233 |
| FLJ43315 | asparagine synthetase pseudogene | 1.8637 | 0.0262 | 71.2602 |
| RSPH6A | radial spoke head 6 homolog A (Chlamydomonas) | 1.3007 | 0.0246 | 52.8400 |
| HIST2H2BA | histone cluster 2, H2ba (pseudogene) | 1.0447 | 0.0244 | 42.8896 |
| ACTR3BP5 | ARP3 actin-related protein 3 homolog B (yeast) pseudogene 5 | 3.0944 | 0.0226 | 137.1385 |
| HRASLS | HRAS-like suppressor | 1.1798 | 0.0213 | 55.4378 |
| UBTFL1 | upstream binding transcription factor, RNA polymerase I-like 1 | 1.3919 | 0.0210 | 66.1984 |
| MIR941-2 | microRNA 941-2 | 2.0759 | 0.0192 | 107.9485 |
| OR4Q3 | olfactory receptor, family 4, subfamily Q, member 3 | 1.3253 | 0.0192 | 68.9132 |
| POM121L4P | POM121 transmembrane nucleoporin-like 4 pseudogene | 1.2622 | 0.0192 | 65.6346 |
| NANOS2 | nanos homolog 2 (Drosophila) | 2.7115 | 0.0179 | 151.0707 |
| FAM157B | family with sequence similarity 157, member B | 2.6532 | 0.0167 | 159.1932 |
| LOC100499194 | uncharacterized LOC100499194 | 1.9980 | 0.0151 | 132.0690 |
| ASIP | agouti signaling protein | 1.9229 | 0.0138 | 138.8748 |
| KRT18P55 | keratin 18 pseudogene 55 | 1.3164 | 0.0121 | 109.2366 |
| GALR3 | galanin receptor 3 | 1.0220 | 0.0121 | 84.8071 |
| OR10G2 | olfactory receptor, family 10, subfamily G, member 2 | 1.6017 | 0.0118 | 135.7959 |
| ASCL5 | achaete-scute complex homolog 5 (Drosophila) | 1.2029 | 0.0115 | 104.2497 |
| PCDHA4 | protocadherin alpha 4 | 1.3292 | 0.0110 | 120.5510 |
| KRTAP5-3 | keratin associated protein 5-3 | 1.1083 | 0.0108 | 102.9140 |
| OR2W3 | olfactory receptor, family 2, subfamily W, member 3 | 1.2481 | 0.0100 | 124.8136 |
| LOC100289656 | Dexi homolog (mouse) pseudogene | 4.0363 | 0.0092 | 437.2627 |
| OR1F2P | olfactory receptor, family 1, subfamily F, member 2 | 1.1778 | 0.0079 | 148.1744 |
| KRTAP1-1 | keratin associated protein 1-1 | 1.0547 | 0.0072 | 146.9110 |
| PRAMEF10 | PRAME family member 10 | 1.0659 | 0.0067 | 159.8898 |
| OR1D2 | olfactory receptor, family 1, subfamily D, member 2 | 2.0563 | 0.0049 | 422.0767 |
| PENK | proenkephalin | 1.8458 | 0.0033 | 553.7289 |
| CT45A3 | cancer/testis antigen family 45, member A3 | 1.5897 | 0.0026 | 619.9677 |
| OR5C1 | olfactory receptor, family 5, subfamily C, member 1 | 2.6229 | 0.0021 | 1278.6548 |
| GJD2 | gap junction protein, delta 2, 36kDa | 1.3317 | 0.0018 | 741.9443 |
| YBX3P1 | Y box binding protein 3 pseudogene 1 | 1.2856 | 0.0015 | 835.6354 |
| CT45A4 | cancer/testis antigen family 45, member A4 | 1.4586 | 0.0013 | 1137.7427 |

DEGs: differentially expressed genes

**Table S3 DEGs reduced in children with stunting compared to adults**

| genename | description | meanfpkm_stunted | meanfpkm_adult | ratiostuntedadult |
| --- | --- | --- | --- | --- |
| MTRNR2L2 | MT-RNR2-like 2 | 6980.8535 | 39332.4922 | 0.1775 |
| MTRNR2L8 | MT-RNR2-like 8 | 2464.3904 | 11676.7227 | 0.2111 |
| IGLL5 | immunoglobulin lambda-like polypeptide 5 | 1963.0981 | 7615.7827 | 0.2578 |
| RNA18S5 | RNA, 18S ribosomal 5 | 817.8422 | 6513.3716 | 0.1256 |
| PHGR1 | proline/histidine/glycine-rich 1 | 1980.1025 | 6488.0039 | 0.3052 |
| RNA28S5 | RNA, 28S ribosomal 5 | 1104.9346 | 4299.6157 | 0.2570 |
| IGJ | immunoglobulin J polypeptide, linker protein for immunoglobulin alpha and mu polypeptides | 624.6829 | 1594.1505 | 0.3919 |
| MTRNR2L6 | MT-RNR2-like 6 | 129.4386 | 948.8513 | 0.1364 |
| MTRNR2L3 | MT-RNR2-like 3 | 14.8025 | 547.2905 | 0.0270 |
| MTRNR2L10 | MT-RNR2-like 10 | 81.2495 | 521.9520 | 0.1557 |
| TM4SF4 | transmembrane 4 L six family member 4 | 203.9861 | 483.0249 | 0.4223 |
| PGC | progastricsin (pepsinogen C) | 42.0017 | 415.2659 | 0.1011 |
| TFF2 | trefoil factor 2 | 60.0375 | 254.8054 | 0.2356 |
| LINC01133 | uncharacterized LOC100505633 | 51.3078 | 218.3156 | 0.2350 |
| HBA1 | hemoglobin, alpha 1 | 56.9778 | 196.6972 | 0.2897 |
| HBA2 | hemoglobin, alpha 2 | 65.0930 | 193.1615 | 0.3370 |
| CCDC152 | coiled-coil domain containing 152 | 2.7319 | 180.0028 | 0.0152 |
| MUC6 | mucin 6, oligomeric mucus/gel-forming | 15.6554 | 160.0644 | 0.0978 |
| RPS17 | ribosomal protein S17 | 0.0792 | 138.0708 | 0.0006 |
| CYP2C18 | cytochrome P450, family 2, subfamily C, polypeptide 18 | 49.3763 | 119.0882 | 0.4146 |
| NPIPB5 | nuclear pore complex interacting protein related gene | 45.3751 | 105.2197 | 0.4312 |
| LEPR | leptin receptor | 2.6832 | 89.4703 | 0.0300 |
| RAPGEFL1 | Rap guanine nucleotide exchange factor (GEF)-like 1 | 32.5293 | 87.0138 | 0.3738 |
| SERPINB9 | serpin peptidase inhibitor, clade B (ovalbumin), member 9 | 25.0215 | 86.8841 | 0.2880 |
| SARM1 | sterile alpha and TIR motif containing 1 | 2.4227 | 82.6362 | 0.0293 |
| EPHX1 | epoxide hydrolase 1, microsomal (xenobiotic) | 27.6927 | 80.0592 | 0.3459 |
| VIPR1 | vasoactive intestinal peptide receptor 1 | 31.8522 | 78.3977 | 0.4063 |
| POLR3H | polymerase (RNA) III (DNA directed) polypeptide H (22.9kD) | 4.8893 | 71.9428 | 0.0680 |
| MGST1 | microsomal glutathione S-transferase 1 | 27.9119 | 68.3941 | 0.4081 |
| C6orf58 | chromosome 6 open reading frame 58 | 8.9993 | 67.2510 | 0.1338 |
| LOC100133286 | uncharacterized LOC100133286 | 0.9825 | 66.9346 | 0.0147 |
| NME7 | NME/NM23 family member 7 | 1.8725 | 66.8885 | 0.0280 |
| MT1F | metallothionein 1F | 26.4492 | 65.7246 | 0.4024 |
| HSD11B2 | hydroxysteroid (11-beta) dehydrogenase 2 | 26.3522 | 62.1554 | 0.4240 |
| TAPSAR1 | uncharacterized LOC100507463 | 24.1932 | 61.1390 | 0.3957 |
| ANKRD19P | ankyrin repeat domain 19, pseudogene | 1.1968 | 54.7321 | 0.0219 |
| TIAF1 | TGFB1-induced anti-apoptotic factor 1 | 22.9553 | 53.5582 | 0.4286 |
| LOC613037 | nuclear pore complex interacting protein pseudogene | 22.0697 | 52.7579 | 0.4183 |
| SNX22 | sorting nexin 22 | 7.0347 | 51.9892 | 0.1353 |
| MINOS1-NBL1 | C1orf151-NBL1 readthrough | 0.9620 | 50.9118 | 0.0189 |
| NEK5 | NIMA-related kinase 5 | 6.7731 | 50.4856 | 0.1342 |
| CAPN12 | calpain 12 | 5.0276 | 48.7362 | 0.1032 |
| CGREF1 | cell growth regulator with EF-hand domain 1 | 15.3303 | 47.9682 | 0.3196 |
| ADH6 | alcohol dehydrogenase 6 (class V) | 17.1215 | 47.5146 | 0.3603 |
| UGT1A6 | UDP glucuronosyltransferase 1 family, polypeptide A6 | 13.7520 | 46.7169 | 0.2944 |
| SLC4A7 | solute carrier family 4, sodium bicarbonate cotransporter, member 7 | 18.3356 | 46.3164 | 0.3959 |
| MXD3 | MAX dimerization protein 3 | 12.7931 | 44.7367 | 0.2860 |
| ANXA10 | annexin A10 | 18.5958 | 43.5362 | 0.4271 |
| SLC46A3 | solute carrier family 46, member 3 | 18.0664 | 42.2744 | 0.4274 |
| CCL15-CCL14 | CCL15-CCL14 readthrough (non-protein coding) | 14.3173 | 38.5244 | 0.3716 |
| SCTR | secretin receptor | 13.6768 | 37.6079 | 0.3637 |
| GABRE | gamma-aminobutyric acid (GABA) A receptor, epsilon | 13.7290 | 36.8941 | 0.3721 |
| PDIA2 | protein disulfide isomerase family A, member 2 | 15.2541 | 36.3051 | 0.4202 |
| SORD | sorbitol dehydrogenase | 13.3715 | 36.2000 | 0.3694 |
| LOC284889 | uncharacterized LOC284889 | 1.5437 | 35.5315 | 0.0434 |
| SULT1E1 | sulfotransferase family 1E, estrogen-preferring, member 1 | 11.4807 | 33.5372 | 0.3423 |
| NT5E | 5'-nucleotidase, ecto (CD73) | 11.0580 | 32.8597 | 0.3365 |
| GP2 | glycoprotein 2 (zymogen granule membrane) | 12.4697 | 32.8305 | 0.3798 |
| NAT2 | N-acetyltransferase 2 (arylamine N-acetyltransferase) | 13.2583 | 30.9418 | 0.4285 |
| AZGP1 | alpha-2-glycoprotein 1, zinc-binding | 11.9017 | 30.0336 | 0.3963 |
| CBR3-AS1 | CBR3 antisense RNA 1 | 3.7678 | 29.6990 | 0.1269 |
| BSCL2 | Berardinelli-Seip congenital lipodystrophy 2 (seipin) | 11.4822 | 27.8192 | 0.4127 |
| LINC00987 | uncharacterized LOC100499405 | 1.4275 | 27.1985 | 0.0525 |
| ZNF585B | zinc finger protein 585B | 2.5646 | 26.0559 | 0.0984 |
| LENG9 | leukocyte receptor cluster (LRC) member 9 | 9.4824 | 25.2403 | 0.3757 |
| CHAD | chondroadherin | 7.3383 | 24.9233 | 0.2944 |
| ANKHD1-EIF4EBP3 | ANKHD1-EIF4EBP3 readthrough | 9.0292 | 22.9192 | 0.3940 |
| TMEM209 | transmembrane protein 209 | 8.5332 | 22.6985 | 0.3759 |
| ASPDH | aspartate dehydrogenase domain containing | 7.1259 | 22.2390 | 0.3204 |
| USP2 | ubiquitin specific peptidase 2 | 9.5571 | 22.0962 | 0.4325 |
| MSMB | microseminoprotein, beta- | 8.5400 | 21.5621 | 0.3961 |
| TMEM120B | transmembrane protein 120B | 8.0424 | 21.2385 | 0.3787 |
| PHKG1 | phosphorylase kinase, gamma 1 (muscle) | 1.6351 | 21.1410 | 0.0773 |
| LINC01089 | uncharacterized LOC338799 | 7.0400 | 20.5228 | 0.3430 |
| BLOC1S1-RDH5 | BLOC1S1-RDH5 readthrough | 4.5259 | 19.9223 | 0.2272 |
| FAM151A | family with sequence similarity 151, member A | 1.3502 | 19.6915 | 0.0686 |
| LOC100507537 | uncharacterized LOC100507537 | 7.7771 | 18.8113 | 0.4134 |
| FUZ | fuzzy planar cell polarity protein | 5.4908 | 18.5810 | 0.2955 |
| TPSG1 | tryptase gamma 1 | 5.6212 | 18.3272 | 0.3067 |
| MSLN | mesothelin | 7.4847 | 17.3592 | 0.4312 |
| ATP1A1-AS1 | ATP1A1 opposite strand | 2.3875 | 17.3528 | 0.1376 |
| PSMC3IP | PSMC3 interacting protein | 2.5398 | 17.2351 | 0.1474 |
| LOC401242 | uncharacterized LOC401242 | 0.2375 | 16.9708 | 0.0140 |
| RPL19P12 | ribosomal protein L19 pseudogene 12 | 4.3163 | 16.9377 | 0.2548 |
| PEX10 | peroxisomal biogenesis factor 10 | 4.9305 | 16.8169 | 0.2932 |
| CYP1A1 | cytochrome P450, family 1, subfamily A, polypeptide 1 | 2.0815 | 16.8100 | 0.1238 |
| HOGA1 | 4-hydroxy-2-oxoglutarate aldolase 1 | 5.6771 | 16.7346 | 0.3392 |
| ATP6V1C2 | ATPase, H+ transporting, lysosomal 42kDa, V1 subunit C2 | 0.6920 | 16.6869 | 0.0415 |
| C5orf45 | chromosome 5 open reading frame 45 | 1.9649 | 16.2110 | 0.1212 |
| FOXH1 | forkhead box H1 | 0.8376 | 15.5674 | 0.0538 |
| LDLRAD2 | low density lipoprotein receptor class A domain containing 2 | 5.8092 | 14.3562 | 0.4046 |
| SLC23A3 | solute carrier family 23 (nucleobase transporters), member 3 | 4.4049 | 14.2903 | 0.3082 |
| KDM4A-AS1 | KDM4A antisense RNA 1 | 5.5341 | 14.1992 | 0.3897 |
| FAM13A-AS1 | FAM13A antisense RNA 1 | 2.4175 | 14.1469 | 0.1709 |
| PREPL | prolyl endopeptidase-like | 4.2266 | 13.8854 | 0.3044 |
| ZSWIM8-AS1 | ZSWIM8 antisense RNA 1 | 4.0517 | 13.6610 | 0.2966 |
| FMO4 | flavin containing monooxygenase 4 | 5.5712 | 13.1956 | 0.4222 |
| PRR4 | proline rich 4 (lacrimal) | 0.8119 | 12.9526 | 0.0627 |
| TBC1D3C | TBC1 domain family, member 3C | 5.2136 | 12.8018 | 0.4073 |
| CHRNE | cholinergic receptor, nicotinic, epsilon (muscle) | 0.3397 | 12.4059 | 0.0274 |
| BBIP1 | BBSome interacting protein 1 | 3.9437 | 12.3792 | 0.3186 |
| SETD6 | SET domain containing 6 | 2.2119 | 12.2315 | 0.1808 |
| CPNE6 | copine VI (neuronal) | 3.3286 | 12.1551 | 0.2738 |
| PCGF2 | polycomb group ring finger 2 | 3.0886 | 11.6687 | 0.2647 |
| CERS6-AS1 | CERS6 antisense RNA 1 | 3.0003 | 11.5915 | 0.2588 |
| AGAP11 | ankyrin repeat and GTPase domain Arf GTPase activating protein 11 | 4.8747 | 11.4051 | 0.4274 |
| PMS2P9 | PMS2 postmeiotic segregation increased 2 (S. cerevisiae) pseudogene | 4.6553 | 11.1964 | 0.4158 |
| GNRHR2 | gonadotropin-releasing hormone (type 2) receptor 2 | 4.7512 | 11.0038 | 0.4318 |
| SLC6A13 | solute carrier family 6 (neurotransmitter transporter, GABA), member 13 | 2.9756 | 10.5477 | 0.2821 |
| CYP4F3 | cytochrome P450, family 4, subfamily F, polypeptide 3 | 2.3908 | 10.4782 | 0.2282 |
| BMS1P4 | BMS1 pseudogene 4 | 4.2271 | 10.3264 | 0.4094 |
| KLKB1 | kallikrein B, plasma (Fletcher factor) 1 | 4.1131 | 10.2674 | 0.4006 |
| GCNT4 | glucosaminyl (N-acetyl) transferase 4, core 2 | 3.8953 | 10.2079 | 0.3816 |
| EMILIN3 | elastin microfibril interfacer 3 | 2.9158 | 10.1346 | 0.2877 |
| ME1 | malic enzyme 1, NADP(+)-dependent, cytosolic | 4.2581 | 10.1054 | 0.4214 |
| TAPT1-AS1 | TAPT1 antisense RNA 1 (head to head) | 1.4759 | 10.0397 | 0.1470 |
| MMP19 | matrix metallopeptidase 19 | 2.2724 | 9.9328 | 0.2288 |
| CNTD1 | cyclin N-terminal domain containing 1 | 1.1186 | 9.8756 | 0.1133 |
| ZNF32-AS1 | ZNF32 antisense RNA 1 | 0.0581 | 9.6895 | 0.0060 |
| LOC100507577 | uncharacterized LOC100507577 | 3.1849 | 9.6600 | 0.3297 |
| SPDEF | SAM pointed domain containing ets transcription factor | 3.2980 | 9.3905 | 0.3512 |
| LHB | luteinizing hormone beta polypeptide | 3.9029 | 9.1962 | 0.4244 |
| COG5 | component of oligomeric golgi complex 5 | 3.4817 | 9.1533 | 0.3804 |
| EIF3CL | eukaryotic translation initiation factor 3, subunit C-like | 1.1734 | 8.7726 | 0.1338 |
| PSORS1C3 | psoriasis susceptibility 1 candidate 3 (non-protein coding) | 2.2231 | 8.6374 | 0.2574 |
| GGT3P | gamma-glutamyltransferase 3 pseudogene | 2.4092 | 8.4928 | 0.2837 |
| DOC2A | double C2-like domains, alpha | 2.3478 | 8.4421 | 0.2781 |
| ALDH3A1 | aldehyde dehydrogenase 3 family, member A1 | 2.4069 | 8.2667 | 0.2912 |
| TSIX | TSIX transcript, XIST antisense RNA | 1.7612 | 8.2213 | 0.2142 |
| MAP3K12 | mitogen-activated protein kinase kinase kinase 12 | 2.0419 | 8.1856 | 0.2494 |
| PRSS16 | protease, serine, 16 (thymus) | 2.7992 | 8.0782 | 0.3465 |
| SLC25A5-AS1 | SLC25A5 antisense RNA 1 | 1.3066 | 7.7503 | 0.1686 |
| RUSC1-AS1 | RUSC1 antisense RNA 1 | 1.1346 | 7.6249 | 0.1488 |
| FAM166A | family with sequence similarity 166, member A | 0.8312 | 7.4705 | 0.1113 |
| TFB1M | transcription factor B1, mitochondrial | 3.0239 | 7.4549 | 0.4056 |
| LRAT | lecithin retinol acyltransferase (phosphatidylcholine--retinol O-acyltransferase) | 2.4325 | 7.3951 | 0.3289 |
| S100A1 | S100 calcium binding protein A1 | 2.9119 | 7.3436 | 0.3965 |
| SNRK-AS1 | SNRK antisense RNA 1 | 0.8914 | 6.9751 | 0.1278 |
| WNT7B | wingless-type MMTV integration site family, member 7B | 2.4253 | 6.9423 | 0.3493 |
| PCDP1 | primary ciliary dyskinesia protein 1 | 2.6131 | 6.9003 | 0.3787 |
| SLC5A12 | solute carrier family 5 (sodium/glucose cotransporter), member 12 | 1.3631 | 6.8721 | 0.1983 |
| ASAH2B | N-acylsphingosine amidohydrolase (non-lysosomal ceramidase) 2B | 1.2054 | 6.8392 | 0.1763 |
| PSORS1C1 | psoriasis susceptibility 1 candidate 1 | 1.6385 | 6.7954 | 0.2411 |
| ZACN | zinc activated ligand-gated ion channel | 0.0937 | 6.4982 | 0.0144 |
| LOC100132062 | uncharacterized LOC100132062 | 2.6425 | 6.4959 | 0.4068 |
| LIPF | lipase, gastric | 0.1914 | 6.3774 | 0.0300 |
| KLK1 | kallikrein 1 | 1.6088 | 6.3546 | 0.2532 |
| FTX | FTX transcript, XIST regulator (non-protein coding) | 2.2741 | 6.3121 | 0.3603 |
| PCNA-AS1 | PCNA antisense RNA 1 | 0.7707 | 6.3028 | 0.1223 |
| LOC100129931 | uncharacterized LOC100129931 | 1.3415 | 6.2572 | 0.2144 |
| FGF5 | fibroblast growth factor 5 | 2.4020 | 6.2462 | 0.3846 |
| TCEB3-AS1 | uncharacterized LOC100506963 | 0.8180 | 6.2354 | 0.1312 |
| PXN-AS1 | PXN antisense RNA 1 | 2.2325 | 6.1215 | 0.3647 |
| AKR1B15 | aldo-keto reductase family 1, member B15 | 1.5380 | 5.8777 | 0.2617 |
| CIART | chromosome 1 open reading frame 51 | 2.1259 | 5.8667 | 0.3624 |
| LOC100379224 | uncharacterized LOC100379224 | 0.4341 | 5.6992 | 0.0762 |
| TMEM200B | transmembrane protein 200B | 2.0786 | 5.6626 | 0.3671 |
| PLCH2 | phospholipase C, eta 2 | 2.2119 | 5.6403 | 0.3922 |
| AQP5 | aquaporin 5 | 0.7771 | 5.5251 | 0.1407 |
| CCDC42B | coiled-coil domain containing 42B | 0.8236 | 5.3387 | 0.1543 |
| LOC100507424 | uncharacterized LOC100507424 | 1.6993 | 5.2128 | 0.3260 |
| RNU1-28P | RNA, U1 small nuclear 8 | 1.9881 | 5.0459 | 0.3940 |
| RNU1-27P | RNA, U1 small nuclear 7 | 1.9881 | 5.0459 | 0.3940 |
| RNVU1-18 | RNA, U1 small nuclear 5 | 1.9881 | 5.0459 | 0.3940 |
| RNU1-2 | RNA, U1 small nuclear 2 | 1.9881 | 5.0459 | 0.3940 |
| RNU1-1 | RNA, U1 small nuclear 1 | 1.9881 | 5.0459 | 0.3940 |
| RNU1-3 | RNA, U1 small nuclear 3 | 1.9881 | 5.0459 | 0.3940 |
| RNU1-4 | RNA, U1 small nuclear 4 | 1.9881 | 5.0459 | 0.3940 |
| LBX2 | ladybird homeobox 2 | 1.7300 | 5.0395 | 0.3433 |
| SYNC | syncoilin, intermediate filament protein | 0.7173 | 5.0287 | 0.1426 |
| LINC00659 | long intergenic non-protein coding RNA 659 | 1.7380 | 5.0123 | 0.3467 |
| LSMEM2 | leucine-rich single-pass membrane protein 2 | 0.2480 | 4.8636 | 0.0510 |
| PPP1R36 | protein phosphatase 1, regulatory subunit 36 | 1.9437 | 4.8046 | 0.4046 |
| KIAA0825 | KIAA0825 | 0.9334 | 4.7515 | 0.1964 |
| BGLAP | bone gamma-carboxyglutamate (gla) protein | 1.7122 | 4.7092 | 0.3636 |
| TMEM52 | transmembrane protein 52 | 1.8824 | 4.6123 | 0.4081 |
| STRIP2 | striatin interacting protein 2 | 1.6836 | 4.5921 | 0.3666 |
| AMPD1 | adenosine monophosphate deaminase 1 | 0.6014 | 4.3331 | 0.1388 |
| GGTLC2 | gamma-glutamyltransferase light chain 2 | 1.5946 | 4.3228 | 0.3689 |
| LOC643201 | centrosomal protein 192kDa pseudogene | 1.7944 | 4.3118 | 0.4162 |
| CASKIN1 | CASK interacting protein 1 | 0.0851 | 4.2854 | 0.0199 |
| LY6G5C | lymphocyte antigen 6 complex, locus G5C | 1.4366 | 4.2713 | 0.3363 |
| HIF1A-AS2 | HIF1A antisense RNA 2 | 0.1381 | 4.2285 | 0.0327 |
| GPR65 | G protein-coupled receptor 65 | 1.7571 | 4.2123 | 0.4171 |
| ZNF138 | zinc finger protein 138 | 1.5581 | 4.1803 | 0.3727 |
| KCNJ13 | potassium inwardly-rectifying channel, subfamily J, member 13 | 0.7966 | 4.1605 | 0.1915 |
| SCN3B | sodium channel, voltage-gated, type III, beta subunit | 1.4081 | 4.1305 | 0.3409 |
| MED31 | mediator complex subunit 31 | 1.7541 | 4.0464 | 0.4335 |
| CERKL | ceramide kinase-like | 1.4185 | 4.0174 | 0.3531 |
| MSTO2P | misato homolog 2 pseudogene | 0.9436 | 3.9715 | 0.2376 |
| COMMD3-BMI1 | COMMD3-BMI1 readthrough | 0.2097 | 3.9628 | 0.0529 |
| C21orf15 | chromosome 21 open reading frame 15 | 0.3568 | 3.8554 | 0.0925 |
| GTF2IRD1P1 | GTF2I repeat domain containing 1 pseusogene 1 | 0.0180 | 3.8223 | 0.0047 |
| TMOD1 | tropomodulin 1 | 1.5937 | 3.8172 | 0.4175 |
| MT1A | metallothionein 1A | 0.8749 | 3.7874 | 0.2310 |
| C1orf56 | chromosome 1 open reading frame 56 | 1.6046 | 3.7397 | 0.4291 |
| TCN1 | transcobalamin I (vitamin B12 binding protein, R binder family) | 0.3439 | 3.7228 | 0.0924 |
| SPTLC3 | serine palmitoyltransferase, long chain base subunit 3 | 1.4859 | 3.7208 | 0.3994 |
| SLC6A12 | solute carrier family 6 (neurotransmitter transporter, betaine/GABA), member 12 | 1.0403 | 3.6931 | 0.2817 |
| EMBP1 | embigin pseudogene 1 | 1.3339 | 3.6915 | 0.3613 |
| MED4-AS1 | MED4 antisense RNA 1 | 0.2375 | 3.6482 | 0.0651 |
| SERPINB5 | serpin peptidase inhibitor, clade B (ovalbumin), member 5 | 1.1898 | 3.5392 | 0.3362 |
| HCG4 | HLA complex group 4 (non-protein coding) | 1.0897 | 3.5108 | 0.3104 |
| DPRXP4 | divergent-paired related homeobox pseudogene 4 | 1.2880 | 3.5033 | 0.3676 |
| LYPD1 | LY6/PLAUR domain containing 1 | 0.5761 | 3.4387 | 0.1675 |
| TBC1D3 | TBC1 domain family, member 3 | 0.5051 | 3.4218 | 0.1476 |
| F11-AS1 | uncharacterized LOC285441 | 0.5114 | 3.4118 | 0.1499 |
| CABP7 | calcium binding protein 7 | 0.5032 | 3.3162 | 0.1517 |
| CNTFR | ciliary neurotrophic factor receptor | 0.6819 | 3.2695 | 0.2086 |
| DCST2 | DC-STAMP domain containing 2 | 1.3712 | 3.2585 | 0.4208 |
| DDO | D-aspartate oxidase | 0.8007 | 3.1910 | 0.2509 |
| LIMD1-AS1 | LIMD1 antisense RNA 1 | 0.3515 | 3.0931 | 0.1136 |
| MT1B | metallothionein 1B | 1.2114 | 3.0564 | 0.3963 |
| PIPSL | PIP5K1A and PSMD4-like, pseudogene | 0.7090 | 3.0236 | 0.2345 |
| LOC100287042 | uncharacterized LOC100287042 | 0.9246 | 2.9818 | 0.3101 |
| GGTLC1 | gamma-glutamyltransferase light chain 1 | 1.1097 | 2.8759 | 0.3858 |
| LOC283177 | uncharacterized LOC283177 | 0.3869 | 2.8692 | 0.1349 |
| LINC00261 | long intergenic non-protein coding RNA 261 | 1.0117 | 2.8513 | 0.3548 |
| SPAG5-AS1 | SPAG5 antisense RNA 1 | 1.0831 | 2.8187 | 0.3842 |
| BMP6 | bone morphogenetic protein 6 | 0.5853 | 2.6987 | 0.2169 |
| SIGLEC15 | sialic acid binding Ig-like lectin 15 | 0.9393 | 2.6651 | 0.3524 |
| CSTF3-AS1 | CSTF3 antisense RNA 1 (head to head) | 0.7134 | 2.6623 | 0.2680 |
| LOC554223 | histocompatibility antigen-related | 1.1002 | 2.6426 | 0.4163 |
| FAM13C | family with sequence similarity 13, member C | 0.5759 | 2.6321 | 0.2188 |
| STX19 | syntaxin 19 | 1.1139 | 2.6274 | 0.4239 |
| MRPL42P5 | mitochondrial ribosomal protein L42 pseudogene 5 | 0.7873 | 2.5836 | 0.3047 |
| CNGA1 | cyclic nucleotide gated channel alpha 1 | 0.9958 | 2.5808 | 0.3858 |
| FAM175A | family with sequence similarity 175, member A | 0.8671 | 2.4962 | 0.3474 |
| PPAN-P2RY11 | PPAN-P2RY11 readthrough | 0.1556 | 2.4862 | 0.0626 |
| MT1DP | metallothionein 1D, pseudogene | 0.5942 | 2.4821 | 0.2394 |
| SMG1P1 | smg-1 homolog, phosphatidylinositol 3-kinase-related kinase pseudogene 1 | 0.9573 | 2.4590 | 0.3893 |
| ACPT | acid phosphatase, testicular | 0.3864 | 2.4300 | 0.1590 |
| CDKN2B-AS1 | CDKN2B antisense RNA 1 | 1.0137 | 2.4164 | 0.4195 |
| TMEM132E | transmembrane protein 132E | 0.8290 | 2.3990 | 0.3456 |
| CNDP1 | carnosine dipeptidase 1 (metallopeptidase M20 family) | 0.6388 | 2.3674 | 0.2698 |
| TLX1 | T-cell leukemia homeobox 1 | 0.6586 | 2.3526 | 0.2800 |
| ANKS1B | ankyrin repeat and sterile alpha motif domain containing 1B | 0.4793 | 2.3156 | 0.2070 |
| MS4A15 | membrane-spanning 4-domains, subfamily A, member 15 | 0.6225 | 2.3026 | 0.2704 |
| CHODL | chondrolectin | 0.9612 | 2.2992 | 0.4180 |
| CYP21A2 | cytochrome P450, family 21, subfamily A, polypeptide 2 | 0.6573 | 2.2987 | 0.2859 |
| CCDC73 | coiled-coil domain containing 73 | 0.1122 | 2.2887 | 0.0490 |
| UGT1A7 | UDP glucuronosyltransferase 1 family, polypeptide A7 | 0.2895 | 2.2490 | 0.1287 |
| CXorf65 | chromosome X open reading frame 65 | 0.9061 | 2.2379 | 0.4049 |
| SPDYA | speedy homolog A (Xenopus laevis) | 0.3836 | 2.2123 | 0.1734 |
| B3GAT2 | beta-1,3-glucuronyltransferase 2 (glucuronosyltransferase S) | 0.2695 | 2.1977 | 0.1226 |
| RNVU1-7 | RNA, U1 small nuclear 9 | 0.8324 | 2.1605 | 0.3853 |
| PRR29 | chromosome 17 open reading frame 72 | 0.6892 | 2.1569 | 0.3195 |
| FEV | FEV (ETS oncogene family) | 0.6819 | 2.1446 | 0.3179 |
| OR6W1P | olfactory receptor, family 6, subfamily W, member 1 pseudogene | 0.5731 | 2.1103 | 0.2716 |
| ANKRD20A4 | ankyrin repeat domain 20 family, member A4 | 0.6775 | 2.0990 | 0.3228 |
| GOLGA6L22 | putative golgin subfamily A member 6-like | 0.8220 | 2.0500 | 0.4010 |
| OR2A4 | olfactory receptor, family 2, subfamily A, member 4 | 0.7558 | 2.0495 | 0.3688 |
| FAM66C | family with sequence similarity 66, member C | 0.7147 | 2.0459 | 0.3494 |
| FAM41C | family with sequence similarity 41, member C | 0.7722 | 2.0297 | 0.3804 |
| NEDD8-MDP1 | NEDD8-MDP1 readthrough | 0.5346 | 2.0100 | 0.2660 |
| A4GNT | alpha-1,4-N-acetylglucosaminyltransferase | 0.4868 | 2.0033 | 0.2430 |
| CTAGE15 | CTAGE family, member 15 | 0.6520 | 1.9913 | 0.3274 |
| TEX35 | testis expressed 35 | 0.1861 | 1.9887 | 0.0936 |
| DEFB131 | defensin, beta 131 | 0.4369 | 1.9723 | 0.2215 |
| AQP7P3 | aquaporin 7 pseudogene 3 | 0.5714 | 1.9490 | 0.2932 |
| SERINC4 | serine incorporator 4 | 0.3268 | 1.9141 | 0.1707 |
| LOC100507557 | uncharacterized LOC100507557 | 0.5508 | 1.9069 | 0.2889 |
| FAM25A | family with sequence similarity 25, member A | 0.5346 | 1.8397 | 0.2906 |
| C19orf18 | chromosome 19 open reading frame 18 | 0.7234 | 1.8228 | 0.3969 |
| PLAC8L1 | PLAC8-like 1 | 0.7469 | 1.8072 | 0.4133 |
| MTMR7 | myotubularin related protein 7 | 0.4219 | 1.7097 | 0.2467 |
| FAM74A3 | family with sequence similarity 74, member A3 | 0.6214 | 1.7069 | 0.3640 |
| TAS2R20 | taste receptor, type 2, member 20 | 0.7014 | 1.6869 | 0.4158 |
| LOC100129148 | uncharacterized LOC100129148 | 0.0619 | 1.6856 | 0.0367 |
| TEX29 | testis expressed 29 | 0.4583 | 1.6613 | 0.2759 |
| TNIP3 | TNFAIP3 interacting protein 3 | 0.6115 | 1.5923 | 0.3840 |
| MYOT | myotilin | 0.6051 | 1.5662 | 0.3864 |
| SAP30L-AS1 | uncharacterized LOC386627 | 0.6342 | 1.5274 | 0.4152 |
| SERPINA4 | serpin peptidase inhibitor, clade A (alpha-1 antiproteinase, antitrypsin), member 4 | 0.3988 | 1.5138 | 0.2634 |
| MIR3936 | microRNA 3936 | 0.1975 | 1.5008 | 0.1316 |
| KMO | kynurenine 3-monooxygenase (kynurenine 3-hydroxylase) | 0.4658 | 1.4562 | 0.3199 |
| SCGB1B2P | secretoglobin, family 1B, member 2, pseudogene | 0.6154 | 1.4505 | 0.4243 |
| GPR75-ASB3 | GPR75-ASB3 readthrough | 0.3402 | 1.4346 | 0.2371 |
| C1orf204 | chromosome 1 open reading frame 204 | 0.4142 | 1.4123 | 0.2933 |
| LOC100652999 | uncharacterized LOC100652999 | 0.0586 | 1.3985 | 0.0419 |
| IL24 | interleukin 24 | 0.2146 | 1.3862 | 0.1548 |
| BIVM-ERCC5 | BIVM-ERCC5 readthrough | 0.0449 | 1.3851 | 0.0324 |
| CACTIN-AS1 | CACTIN antisense RNA 1 | 0.4014 | 1.3610 | 0.2949 |
| PRCD | progressive rod-cone degeneration | 0.2071 | 1.3287 | 0.1559 |
| SULT1C3 | sulfotransferase family, cytosolic, 1C, member 3 | 0.3988 | 1.3192 | 0.3023 |
| LINC01192 | cancer/testis antigen 64 (non-protein coding) | 0.2981 | 1.2869 | 0.2317 |
| HABP2 | hyaluronan binding protein 2 | 0.5344 | 1.2854 | 0.4158 |
| HLF | hepatic leukemia factor | 0.5364 | 1.2844 | 0.4177 |
| SNORD17 | small nucleolar RNA, C/D box 17 | 0.4649 | 1.2787 | 0.3636 |
| CLVS1 | clavesin 1 | 0.0195 | 1.2721 | 0.0153 |
| TTBK1 | tau tubulin kinase 1 | 0.4754 | 1.2710 | 0.3740 |
| FTCD | formiminotransferase cyclodeaminase | 0.4229 | 1.2656 | 0.3341 |
| LOC145474 | uncharacterized LOC145474 | 0.4432 | 1.2651 | 0.3503 |
| ANKRD20A11P | ankyrin repeat domain 20 family, member A11, pseudogene | 0.2731 | 1.2564 | 0.2173 |
| LOC646626 | uncharacterized LOC646626 | 0.3302 | 1.2554 | 0.2630 |
| HCG25 | HLA complex group 25 (non-protein coding) | 0.5034 | 1.2531 | 0.4017 |
| HOXA7 | homeobox A7 | 0.3015 | 1.2495 | 0.2413 |
| C6orf163 | chromosome 6 open reading frame 163 | 0.3454 | 1.2446 | 0.2775 |
| SERHL | serine hydrolase-like | 0.1302 | 1.2349 | 0.1054 |
| CC2D2B | coiled-coil and C2 domain containing 2B | 0.1636 | 1.2223 | 0.1338 |
| RPS10-NUDT3 | RPS10-NUDT3 readthrough | 0.0310 | 1.1941 | 0.0260 |
| LINC00883 | uncharacterized LOC344595 | 0.4907 | 1.1892 | 0.4126 |
| BDNF | brain-derived neurotrophic factor | 0.1939 | 1.1838 | 0.1638 |
| SCARNA7 | small Cajal body-specific RNA 7 | 0.2581 | 1.1705 | 0.2205 |
| FAM71F2 | family with sequence similarity 71, member F2 | 0.4437 | 1.1656 | 0.3807 |
| MCM3AP-AS1 | MCM3AP antisense RNA 1 | 0.4110 | 1.1233 | 0.3659 |
| SPTY2D1-AS1 | SPTY2D1 antisense RNA 1 | 0.0515 | 1.1231 | 0.0459 |
| TGIF2-C20orf24 | TGIF2-C20orf24 readthrough | 0.0239 | 1.1174 | 0.0214 |
| SLC47A2 | solute carrier family 47, member 2 | 0.3793 | 1.1174 | 0.3395 |
| DNAJB13 | DnaJ (Hsp40) homolog, subfamily B, member 13 | 0.4092 | 1.1162 | 0.3666 |
| CDNF | cerebral dopamine neurotrophic factor | 0.4510 | 1.1069 | 0.4075 |
| KCTD21-AS1 | uncharacterized LOC100289388 | 0.3103 | 1.1026 | 0.2815 |
| LYPD8 | LY6/PLAUR domain containing 8 | 0.3110 | 1.0959 | 0.2838 |
| SERPINI2 | serpin peptidase inhibitor, clade I (pancpin), member 2 | 0.1876 | 1.0941 | 0.1715 |
| LOC100652770 | uncharacterized LOC100652770 | 0.1629 | 1.0910 | 0.1493 |
| CHIT1 | chitinase 1 (chitotriosidase) | 0.3690 | 1.0831 | 0.3407 |
| DDX11L2 | DEAD/H (Asp-Glu-Ala-Asp/His) box helicase 11 like 2 | 0.2481 | 1.0649 | 0.2330 |
| OR2B6 | olfactory receptor, family 2, subfamily B, member 6 | 0.3925 | 1.0641 | 0.3689 |
| NEK10 | NIMA-related kinase 10 | 0.1849 | 1.0572 | 0.1749 |
| GAS5-AS1 | GAS5 antisense RNA 1 | 0.4337 | 1.0572 | 0.4103 |
| LOC100506124 | uncharacterized LOC100506124 | 0.4447 | 1.0549 | 0.4216 |
| F5 | coagulation factor V (proaccelerin, labile factor) | 0.4214 | 1.0508 | 0.4010 |
| FAM155A | family with sequence similarity 155, member A | 0.3588 | 1.0418 | 0.3444 |
| RFPL3 | ret finger protein-like 3 | 0.1019 | 1.0303 | 0.0989 |
| NUP62CL | nucleoporin 62kDa C-terminal like | 0.3778 | 1.0264 | 0.3681 |
| SNX32 | sorting nexin 32 | 0.3793 | 1.0241 | 0.3704 |

**Table S4 DEGs increased in children with SAM compared to adults**

| genename | description | meanfpkm_sam | meanfpkm_adult | ratiosamadult |
| --- | --- | --- | --- | --- |
| MIR650 | microRNA 650 | 3699.5244 | 1.4726 | 2512.3010 |
| MTRNR2L4 | MT-RNR2-like 4 | 1784.7836 | 3.4556 | 516.4841 |
| RPL13AP5 | ribosomal protein L13a pseudogene 5 | 1151.8955 | 2.9041 | 396.6442 |
| MT1H | metallothionein 1H | 907.1454 | 30.4879 | 29.7542 |
| IGLL3P | immunoglobulin lambda-like polypeptide 3, pseudogene | 464.7431 | 2.9087 | 159.7759 |
| MT1M | metallothionein 1M | 293.5200 | 8.8146 | 33.2992 |
| IGLL1 | immunoglobulin lambda-like polypeptide 1 | 275.8381 | 8.0503 | 34.2645 |
| SAA1 | serum amyloid A1 | 248.7888 | 1.5646 | 159.0096 |
| MT1B | metallothionein 1B | 222.1893 | 3.0564 | 72.6962 |
| S100P | S100 calcium binding protein P | 209.8814 | 2.2521 | 93.1957 |
| KIAA0101 | KIAA0101 | 146.4269 | 5.2669 | 27.8012 |
| PI3 | peptidase inhibitor 3, skin-derived | 122.9114 | 3.0954 | 39.7080 |
| PSCA | prostate stem cell antigen | 102.4343 | 0.8951 | 114.4353 |
| GGTLC2 | gamma-glutamyltransferase light chain 2 | 102.3143 | 4.3228 | 23.6684 |
| DUOXA2 | dual oxidase maturation factor 2 | 97.9040 | 2.7179 | 36.0213 |
| SAA2 | serum amyloid A2 | 89.6533 | 2.0041 | 44.7349 |
| LOC100506385 | uncharacterized LOC100506385 | 80.4881 | 4.2705 | 18.8474 |
| MAN1B1-AS1 | uncharacterized LOC100289341 | 48.8236 | 1.5556 | 31.3849 |
| CC2D2B | coiled-coil and C2 domain containing 2B | 38.3514 | 1.2223 | 31.3762 |
| MIR1273A | microRNA 1273a | 38.1574 | 0.1951 | 195.5503 |
| LINC01021 | uncharacterized LOC643401 | 33.7988 | 1.6615 | 20.3419 |
| MIR5047 | microRNA 5047 | 33.6926 | 0.2128 | 158.3147 |
| LOC349160 | uncharacterized LOC349160 | 32.8712 | 0.0762 | 431.6419 |
| C2orf15 | chromosome 2 open reading frame 15 | 31.0955 | 1.4564 | 21.3508 |
| MTRNR2L5 | MT-RNR2-like 5 | 30.8310 | 0.7038 | 43.8035 |
| LL22NC01-81G9.3 | uncharacterized protein FLJ39582-like | 30.4112 | 0.0795 | 382.5924 |
| DUOXA1 | dual oxidase maturation factor 1 | 29.9914 | 0.7105 | 42.2110 |
| GKN1 | gastrokine 1 | 29.6314 | 0.3997 | 74.1261 |
| CATSPER2P1 | cation channel, sperm associated 2 pseudogene 1 | 25.5760 | 0.9528 | 26.8424 |
| LOC101243545 | uncharacterized LOC101243545 | 25.2021 | 0.4013 | 62.8041 |
| C14orf178 | chromosome 14 open reading frame 178 | 24.9919 | 0.8800 | 28.3999 |
| AARD | alanine and arginine rich domain containing protein | 24.8243 | 0.3228 | 76.8981 |
| KCTD21-AS1 | uncharacterized LOC100289388 | 24.1157 | 1.1026 | 21.8724 |
| NME1-NME2 | NME1-NME2 readthrough | 20.0557 | 0.3267 | 61.3950 |
| MRGPRX3 | MAS-related GPR, member X3 | 20.0376 | 0.8964 | 22.3532 |
| BAIAP2-AS1 | BAIAP2 antisense RNA 1 (head to head) | 18.6248 | 0.9856 | 18.8961 |
| LINC00330 | long intergenic non-protein coding RNA 330 | 17.4212 | 0.7451 | 23.3801 |
| LINC00381 | long intergenic non-protein coding RNA 381 | 17.1464 | 0.4056 | 42.2700 |
| KCNE4 | potassium voltage-gated channel, Isk-related family, member 4 | 16.6662 | 0.8415 | 19.8044 |
| NANOGNB | NANOG neighbor homeobox | 16.4543 | 0.0154 | 1069.5287 |
| KLF3-AS1 | uncharacterized FLJ13197 | 16.3093 | 0.6405 | 25.4629 |
| CEACAM7 | carcinoembryonic antigen-related cell adhesion molecule 7 | 15.9743 | 0.2759 | 57.8994 |
| GKN2 | gastrokine 2 | 15.6976 | 0.3131 | 50.1398 |
| A2M-AS1 | A2M antisense RNA 1 | 15.3555 | 0.5708 | 26.9031 |
| SKP1P2 | S-phase kinase-associated protein 1 pseudogene 2 | 15.2190 | 0.1233 | 123.3977 |
| TMEM183B | transmembrane protein 183B | 14.0474 | 0.4962 | 28.3126 |
| SNORA31 | small nucleolar RNA, H/ACA box 31 | 13.5864 | 0.5574 | 24.3731 |
| SNORA61 | small nucleolar RNA, H/ACA box 61 | 13.1757 | 0.0274 | 480.2363 |
| SHANK2-AS3 | SHANK2 antisense RNA 3 | 12.6021 | 0.3936 | 32.0185 |
| RNY3 | RNA, Ro-associated Y3 | 12.4883 | 0.1859 | 67.1786 |
| ZNF670-ZNF695 | ZNF670-ZNF695 readthrough | 12.3740 | 0.4544 | 27.2341 |
| INO80B-WBP1 | INO80B-WBP1 readthrough | 11.3921 | 0.2356 | 48.3453 |
| LOC646736 | uncharacterized LOC646736 | 11.0631 | 0.0090 | 1232.7449 |
| PRSS1 | protease, serine, 1 (trypsin 1) | 11.0210 | 0.0341 | 323.1708 |
| DPY19L1P1 | dpy-19-like 1 pseudogene 1 (C. elegans) | 10.5962 | 0.4846 | 21.8652 |
| IL31RA | interleukin 31 receptor A | 10.3048 | 0.1523 | 67.6575 |
| GSTTP2 | glutathione S-transferase theta pseudogene 2 | 10.1717 | 0.1279 | 79.4980 |
| LRRC2 | leucine rich repeat containing 2 | 10.0021 | 0.3723 | 26.8653 |
| RFPL1S | RFPL1 antisense RNA 1 | 9.8167 | 0.4459 | 22.0155 |
| PIN4P1 | protein (peptidylprolyl cis/trans isomerase) NIMA-interacting, 4 pseudogene 1 | 9.7440 | 0.1756 | 55.4771 |
| PCAT18 | uncharacterized LOC728606 | 8.9026 | 0.4544 | 19.5938 |
| LINC00114 | long intergenic non-protein coding RNA 114 | 8.8240 | 0.1595 | 55.3276 |
| CNGB1 | cyclic nucleotide gated channel beta 1 | 8.7898 | 0.0979 | 89.7384 |
| SCARNA22 | small Cajal body-specific RNA 22 | 8.6064 | 0.2023 | 42.5413 |
| SHISA2 | shisa homolog 2 (Xenopus laevis) | 8.5419 | 0.2618 | 32.6282 |
| GCSAML | germinal center-associated, signaling and motility-like | 8.5267 | 0.0318 | 268.1774 |
| RAB4B-EGLN2 | RAB4B-EGLN2 readthrough (non-protein coding) | 8.4574 | 0.1236 | 68.4311 |
| LOC100131626 | uncharacterized LOC100131626 | 8.4124 | 0.3113 | 27.0249 |
| LOC400548 | uncharacterized LOC400548 | 8.2857 | 0.1756 | 47.1741 |
| PDZD9 | PDZ domain containing 9 | 8.2733 | 0.1482 | 55.8235 |
| LINC00637 | long intergenic non-protein coding RNA 637 | 8.2476 | 0.1644 | 50.1805 |
| CXorf31 | chromosome X open reading frame 31 | 8.1076 | 0.1851 | 43.7946 |
| SLC30A2 | solute carrier family 30 (zinc transporter), member 2 | 7.9540 | 0.3682 | 21.6022 |
| MIR4516 | microRNA 4516 | 7.8198 | 0.0600 | 130.3294 |
| NAP1L6 | nucleosome assembly protein 1-like 6 | 7.7540 | 0.3249 | 23.8680 |
| LOC100507462 | uncharacterized LOC100507462 | 7.7238 | 0.2326 | 33.2115 |
| PGA4 | pepsinogen 4, group I (pepsinogen A) | 7.3755 | 0.1769 | 41.6875 |
| LOC100131655 | uncharacterized LOC100131655 | 7.2769 | 0.2746 | 26.4985 |
| CCL16 | chemokine (C-C motif) ligand 16 | 6.8264 | 0.3174 | 21.5049 |
| MT1JP | metallothionein 1J, pseudogene | 6.8126 | 0.1172 | 58.1383 |
| NRSN1 | neurensin 1 | 6.6531 | 0.0646 | 102.9646 |
| OR51E2 | olfactory receptor, family 51, subfamily E, member 2 | 6.2940 | 0.2933 | 21.4570 |
| BEND2 | BEN domain containing 2 | 6.0950 | 0.2946 | 20.6880 |
| TMPRSS11B | transmembrane protease, serine 11B | 5.8302 | 0.0556 | 104.7831 |
| OTX2-AS1 | OTX2 antisense RNA 1 (head to head) | 5.7933 | 0.1062 | 54.5749 |
| UBE2F-SCLY | UBE2F-SCLY readthrough | 5.7862 | 0.0390 | 148.4615 |
| SLC5A10 | solute carrier family 5 (sodium/glucose cotransporter), member 10 | 5.7814 | 0.2728 | 21.1913 |
| MROH7-TTC4 | HEATR8-TTC4 readthrough | 5.7645 | 0.2795 | 20.6254 |
| FKBP1AP1 | FK506 binding protein 1A, 12kDa pseudogene 1 | 5.6902 | 0.2151 | 26.4505 |
| PBOV1 | prostate and breast cancer overexpressed 1 | 5.6705 | 0.2844 | 19.9413 |
| GP6 | glycoprotein VI (platelet) | 5.4664 | 0.2423 | 22.5599 |
| MYL10 | myosin, light chain 10, regulatory | 5.3179 | 0.0274 | 193.8284 |
| FCGR1C | Fc fragment of IgG, high affinity Ic, receptor (CD64), pseudogene | 5.2955 | 0.0651 | 81.3085 |
| RNY1 | RNA, Ro-associated Y1 | 5.1855 | 0.1433 | 36.1777 |
| SYCP3 | synaptonemal complex protein 3 | 5.0569 | 0.2208 | 22.9058 |
| PLCXD3 | phosphatidylinositol-specific phospholipase C, X domain containing 3 | 4.9195 | 0.1197 | 41.0838 |
| PAPL | iron/zinc purple acid phosphatase-like protein | 4.7405 | 0.0944 | 50.2387 |
| C12orf77 | chromosome 12 open reading frame 77 | 4.7081 | 0.1418 | 33.2036 |
| LINC00908 | uncharacterized LOC284276 | 4.7069 | 0.1385 | 33.9943 |
| LINC00948 | uncharacterized LOC100507027 | 4.6210 | 0.0905 | 51.0530 |
| DEFB4A | defensin, beta 4A | 4.4452 | 0.1215 | 36.5747 |
| LINC01010 | uncharacterized LOC154092 | 4.4250 | 0.0262 | 169.1912 |
| EGFLAM-AS4 | EGFLAM antisense RNA 4 | 4.3669 | 0.1049 | 41.6404 |
| IGFBP7-AS1 | uncharacterized LOC255130 | 4.3421 | 0.1436 | 30.2399 |
| SNORA45B | small nucleolar RNA, H/ACA box 45 | 4.2286 | 0.0749 | 56.4775 |
| SNORA76C | small nucleolar RNA, H/ACA box 76 | 4.1762 | 0.0962 | 43.4324 |
| LINC00331 | long intergenic non-protein coding RNA 331 | 4.1657 | 0.1110 | 37.5203 |
| MIR570 | microRNA 570 | 4.1464 | 0.0423 | 98.0065 |
| ATP5L2 | ATP synthase, H+ transporting, mitochondrial Fo complex, subunit G2 | 4.1431 | 0.1792 | 23.1160 |
| SAA4 | serum amyloid A4, constitutive | 4.0617 | 0.1241 | 32.7283 |
| NEUROD2 | neuronal differentiation 2 | 4.0069 | 0.1256 | 31.8917 |
| HLA-DPB2 | major histocompatibility complex, class II, DP beta 2 (pseudogene) | 3.9855 | 0.0633 | 62.9286 |
| TEX101 | testis expressed 101 | 3.9760 | 0.0626 | 63.5501 |
| SSX5 | synovial sarcoma, X breakpoint 5 | 3.9607 | 0.0092 | 429.0774 |
| LINC00466 | long intergenic non-protein coding RNA 466 | 3.8843 | 0.0415 | 93.5106 |
| MKNK1-AS1 | MKNK1 antisense RNA 1 | 3.8167 | 0.1928 | 19.7939 |
| ZNF876P | zinc finger protein 876, pseudogene | 3.8014 | 0.2026 | 18.7665 |
| DBIL5P2 | diazepam binding inhibitor-like 5 pseudogene 2 | 3.7033 | 0.1659 | 22.3230 |
| LOC729987 | uncharacterized LOC729987 | 3.6448 | 0.0897 | 40.6131 |
| PRR23A | proline rich 23A | 3.6362 | 0.1528 | 23.7939 |
| SNORA18 | small nucleolar RNA, H/ACA box 18 | 3.6360 | 0.0541 | 67.2048 |
| C5orf60 | chromosome 5 open reading frame 60 | 3.5836 | 0.1195 | 29.9913 |
| BMS1P17 | ribosome biogenesis protein BMS1 homolog pseudogene | 3.5493 | 0.0805 | 44.0835 |
| SNORA7B | small nucleolar RNA, H/ACA box 7B | 3.5186 | 0.0774 | 45.4385 |
| XKR5 | XK, Kell blood group complex subunit-related family, member 5 | 3.3562 | 0.0110 | 304.3987 |
| LOC100131347 | RAD52 motif 1 pseudogene | 3.3157 | 0.1144 | 28.9939 |
| LINC01136 | uncharacterized LOC730227 | 3.3060 | 0.1033 | 31.9931 |
| WWTR1-AS1 | WWTR1 antisense RNA 1 | 3.1507 | 0.1464 | 21.5198 |
| SVOP | SV2 related protein homolog (rat) | 3.1305 | 0.1367 | 22.9059 |
| NOX4 | NADPH oxidase 4 | 3.0517 | 0.1487 | 20.5198 |
| DPPA4 | developmental pluripotency associated 4 | 3.0200 | 0.0292 | 103.3158 |
| GPR12 | G protein-coupled receptor 12 | 2.9431 | 0.0544 | 54.1418 |
| LOC644172 | mitogen-activated protein kinase 8 interacting protein 1 pseudogene | 2.9148 | 0.1428 | 20.4086 |
| LINC00629 | long intergenic non-protein coding RNA 629 | 2.9000 | 0.0410 | 70.6875 |
| ADORA2B | adenosine A2b receptor | 2.8421 | 0.0685 | 41.5144 |
| LOC100507156 | uncharacterized LOC100507156 | 2.8186 | 0.1251 | 22.5255 |
| LOC100129636 | uncharacterized LOC100129636 | 2.7700 | 0.0351 | 78.8540 |
| GNG8 | guanine nucleotide binding protein (G protein), gamma 8 | 2.6140 | 0.0815 | 32.0591 |
| DISC2 | disrupted in schizophrenia 2 (non-protein coding) | 2.5664 | 0.0805 | 31.8760 |
| SNORA52 | small nucleolar RNA, H/ACA box 52 | 2.5443 | 0.1110 | 22.9162 |
| C2CD4A | C2 calcium-dependent domain containing 4A | 2.5276 | 0.0903 | 28.0049 |
| SNORA24 | small nucleolar RNA, H/ACA box 24 | 2.5174 | 0.1318 | 19.1007 |
| ANXA2P2 | annexin A2 pseudogene 2 | 2.4407 | 0.0213 | 114.6842 |
| TMC1 | transmembrane channel-like 1 | 2.3852 | 0.0751 | 31.7489 |
| DMBX1 | diencephalon/mesencephalon homeobox 1 | 2.3643 | 0.0415 | 56.9180 |
| EEF1DP3 | eukaryotic translation elongation factor 1 delta pseudogene 3 | 2.3374 | 0.0613 | 38.1414 |
| LOC157273 | uncharacterized LOC157273 | 2.2729 | 0.0021 | 1108.0179 |
| CLCA4 | chloride channel accessory 4 | 2.2650 | 0.0505 | 44.8401 |
| PATE2 | prostate and testis expressed 2 | 2.2521 | 0.0079 | 283.3341 |
| MMP10 | matrix metallopeptidase 10 (stromelysin 2) | 2.2290 | 0.0815 | 27.3374 |
| LIPE-AS1 | uncharacterized LOC100996307 | 2.2171 | 0.0723 | 30.6626 |
| LOC100505817 | uncharacterized LOC100505817 | 2.2024 | 0.1126 | 19.5656 |
| HTR5AOS | uncharacterized LOC100128264 | 2.1940 | 0.0695 | 31.5749 |
| LINC00568 | long intergenic non-protein coding RNA 568 | 2.1298 | 0.0418 | 50.9575 |
| C21orf37 | chromosome 21 open reading frame 37 | 2.0914 | 0.0374 | 55.8669 |
| MIR210 | microRNA 210 | 2.0898 | 0.0823 | 25.3896 |
| LOC100128593 | uncharacterized LOC100128593 | 2.0888 | 0.0618 | 33.8023 |
| FLJ43879 | FLJ43879 protein | 2.0745 | 0.0031 | 674.2203 |
| FTLP10 | ferritin, light polypeptide pseudogene 10 | 2.0743 | 0.0559 | 37.1088 |
| SLC9B1 | solute carrier family 9, subfamily B (NHA1, cation proton antiporter 1), member 1 | 2.0693 | 0.1044 | 19.8285 |
| RHCE | Rh blood group, CcEe antigens | 2.0550 | 0.0762 | 26.9848 |
| CADM2-AS2 | CADM2 antisense RNA 2 | 2.0314 | 0.0085 | 240.0779 |
| PGAM1P5 | phosphoglycerate mutase 1 pseudogene 5 | 2.0290 | 0.0510 | 39.7653 |
| SLC35G3 | solute carrier family 35, member G3 | 2.0014 | 0.0892 | 22.4298 |
| CFHR1 | complement factor H-related 1 | 1.9840 | 0.1005 | 19.7393 |
| LINC00889 | uncharacterized LOC158696 | 1.9774 | 0.0856 | 23.0892 |
| RNU6-35P | RNA, U6 small nuclear 35 | 1.9376 | 0.0415 | 46.6464 |
| VSX1 | visual system homeobox 1 | 1.9124 | 0.0592 | 32.2870 |
| LINC01205 | uncharacterized LOC401082 | 1.8805 | 0.0903 | 20.8348 |
| LOC100132078 | uncharacterized LOC100132078 | 1.8643 | 0.0059 | 316.1180 |
| GTF2IRD2P1 | GTF2I repeat domain containing 2 pseudogene 1 | 1.8517 | 0.0290 | 63.9071 |
| CACNG2 | calcium channel, voltage-dependent, gamma subunit 2 | 1.8505 | 0.0977 | 18.9419 |
| MIR205HG | MIR205 host gene (non-protein coding) | 1.7998 | 0.0405 | 44.4245 |
| SNORA40 | small nucleolar RNA, H/ACA box 40 | 1.7407 | 0.0703 | 24.7766 |
| CCDC148 | coiled-coil domain containing 148 | 1.7374 | 0.0808 | 21.5104 |
| LYPD4 | LY6/PLAUR domain containing 4 | 1.7329 | 0.0669 | 25.8933 |
| RNU4-1 | RNA, U4 small nuclear 1 | 1.7300 | 0.0236 | 73.3370 |
| FAM138D | family with sequence similarity 138, member D | 1.6376 | 0.0859 | 19.0648 |
| CHIAP2 | chitinase, acidic pseudogene 2 | 1.5755 | 0.0174 | 90.3582 |
| NKPD1 | NTPase, KAP family P-loop domain containing 1 | 1.5731 | 0.0426 | 36.9583 |
| SPANXA2-OT1 | SPANXA2 overlapping transcript 1 (non-protein coding) | 1.5664 | 0.0136 | 115.2655 |
| LINC00410 | long intergenic non-protein coding RNA 410 | 1.5424 | 0.0605 | 25.4885 |
| C20orf203 | chromosome 20 open reading frame 203 | 1.5138 | 0.0459 | 32.9824 |
| PCDHB19P | protocadherin beta 19 pseudogene | 1.4955 | 0.0474 | 31.5263 |
| KLK4 | kallikrein-related peptidase 4 | 1.4924 | 0.0379 | 39.3263 |
| LINC01194 | cancer/testis antigen 49 (non-protein coding) | 1.4790 | 0.0749 | 19.7544 |
| NFE4 | transcription factor NF-E4 | 1.4757 | 0.0151 | 97.5472 |
| LINC01158 | uncharacterized LOC100506421 | 1.4750 | 0.0136 | 108.5377 |
| FAM138C | family with sequence similarity 138, member C | 1.4655 | 0.0785 | 18.6776 |
| TLR8-AS1 | TLR8 antisense RNA 1 | 1.4302 | 0.0264 | 54.1546 |
| ADIPOQ-AS1 | ADIPOQ antisense RNA 1 | 1.4136 | 0.0133 | 106.0179 |
| LINC00927 | uncharacterized LOC283688 | 1.4129 | 0.0236 | 59.8929 |
| GRIN3B | glutamate receptor, ionotropic, N-methyl-D-aspartate 3B | 1.3986 | 0.0515 | 27.1365 |
| COL4A2-AS1 | COL4A2 antisense RNA 1 | 1.3950 | 0.0108 | 129.5357 |
| KRT16 | keratin 16 | 1.3893 | 0.0231 | 60.2024 |
| LINC00967 | uncharacterized LOC100505659 | 1.3893 | 0.0528 | 26.3020 |
| TRIM60 | tripartite motif containing 60 | 1.3826 | 0.0287 | 48.1448 |
| RD3 | retinal degeneration 3 | 1.3702 | 0.0733 | 18.6851 |
| LEP | leptin | 1.3657 | 0.0328 | 41.6116 |
| CYP8B1 | cytochrome P450, family 8, subfamily B, polypeptide 1 | 1.3574 | 0.0577 | 23.5279 |
| PF4 | platelet factor 4 | 1.3507 | 0.0249 | 54.3071 |
| ASIP | agouti signaling protein | 1.3400 | 0.0138 | 96.7778 |
| LOC284578 | uncharacterized LOC284578 | 1.3305 | 0.0541 | 24.5917 |
| LHFPL5 | lipoma HMGIC fusion partner-like 5 | 1.3186 | 0.0567 | 23.2689 |
| SLC22A9 | solute carrier family 22 (organic anion transporter), member 9 | 1.3017 | 0.0377 | 34.5340 |
| DIRC1 | disrupted in renal carcinoma 1 | 1.2638 | 0.0049 | 259.4135 |
| LOC286177 | uncharacterized LOC286177 | 1.2267 | 0.0349 | 35.1765 |
| LOC286359 | uncharacterized LOC286359 | 1.2074 | 0.0431 | 28.0285 |
| NLRP8 | NLR family, pyrin domain containing 8 | 1.2036 | 0.0538 | 22.3520 |
| DNAJC5G | DnaJ (Hsp40) homolog, subfamily C, member 5 gamma | 1.2005 | 0.0477 | 25.1713 |
| FRG2C | FSHD region gene 2 family, member C | 1.1921 | 0.0477 | 24.9965 |
| FAM138E | family with sequence similarity 138, member E | 1.1757 | 0.0518 | 22.6994 |
| RHPN1-AS1 | RHPN1 antisense RNA 1 (head to head) | 1.1555 | 0.0277 | 41.7255 |
| APOC4 | apolipoprotein C-IV | 1.1436 | 0.0164 | 69.6864 |
| RAX2 | retina and anterior neural fold homeobox 2 | 1.0902 | 0.0033 | 327.0715 |
| LINC01191 | uncharacterized LOC440900 | 1.0567 | 0.0564 | 18.7318 |
| LINC00221 | long intergenic non-protein coding RNA 221 | 1.0388 | 0.0172 | 60.4680 |
| ALPP | alkaline phosphatase, placental | 1.0307 | 0.0264 | 39.0270 |
| SNORA41 | small nucleolar RNA, H/ACA box 41 | 1.0276 | 0.0282 | 36.4338 |
| SNORD3B-2 | small nucleolar RNA, C/D box 3B-2 | 1.0112 | 0.0172 | 58.8603 |
| SNORD3B-1 | small nucleolar RNA, C/D box 3B-1 | 1.0112 | 0.0172 | 58.8603 |
| CLEC4C | C-type lectin domain family 4, member C | 1.0069 | 0.0218 | 46.1992 |

**Table S5 DEGs reduced in children with SAM compared to adults**

| genename | description | meanfpkm_sam | meanfpkm_adult | ratiosamadult |
| --- | --- | --- | --- | --- |
| RNA18S5 | RNA, 18S ribosomal 5 | 6.199 | 6513.372 | 0.001 |
| RNA28S5 | RNA, 28S ribosomal 5 | 7.252 | 4299.616 | 0.002 |
| CYP3A4 | cytochrome P450, family 3, subfamily A, polypeptide 4 | 127.689 | 586.505 | 0.218 |
| MALAT1 | metastasis associated lung adenocarcinoma transcript 1 (non-protein coding) | 16.677 | 514.418 | 0.032 |
| TM4SF4 | transmembrane 4 L six family member 4 | 65.148 | 483.025 | 0.135 |
| SLC26A3 | solute carrier family 26, member 3 | 62.630 | 417.776 | 0.150 |
| TMPRSS15 | transmembrane protease, serine 15 | 95.637 | 393.314 | 0.243 |
| SLC13A2 | solute carrier family 13 (sodium-dependent dicarboxylate transporter), member 2 | 73.623 | 305.913 | 0.241 |
| SI | sucrase-isomaltase (alpha-glucosidase) | 51.545 | 274.831 | 0.188 |
| APOB | apolipoprotein B (including Ag(x) antigen) | 62.302 | 257.743 | 0.242 |
| LINC01133 | uncharacterized LOC100505633 | 54.734 | 218.316 | 0.251 |
| LUC7L3 | LUC7-like 3 (S. cerevisiae) | 48.235 | 193.418 | 0.249 |
| HBA2 | hemoglobin, alpha 2 | 30.771 | 193.162 | 0.159 |
| MUC6 | mucin 6, oligomeric mucus/gel-forming | 37.940 | 160.064 | 0.237 |
| UGT1A1 | UDP glucuronosyltransferase 1 family, polypeptide A1 | 18.438 | 152.675 | 0.121 |
| SLC11A2 | solute carrier family 11 (proton-coupled divalent metal ion transporters), member 2 | 36.519 | 147.935 | 0.247 |
| RPS17 | ribosomal protein S17 | 4.867 | 138.071 | 0.035 |
| CYP4F2 | cytochrome P450, family 4, subfamily F, polypeptide 2 | 31.851 | 126.370 | 0.252 |
| NCOR1 | nuclear receptor corepressor 1 | 15.751 | 112.274 | 0.140 |
| NPIPB5 | nuclear pore complex interacting protein related gene | 8.643 | 105.220 | 0.082 |
| ADH4 | alcohol dehydrogenase 4 (class II), pi polypeptide | 18.225 | 104.668 | 0.174 |
| SLC28A2 | solute carrier family 28 (sodium-coupled nucleoside transporter), member 2 | 15.652 | 103.626 | 0.151 |
| SRSF11 | serine/arginine-rich splicing factor 11 | 24.393 | 96.382 | 0.253 |
| CYP2B7P | cytochrome P450, family 2, subfamily B, polypeptide 7 pseudogene 1 | 14.062 | 85.958 | 0.164 |
| RBM25 | RNA binding motif protein 25 | 13.963 | 84.875 | 0.165 |
| FOXK1 | forkhead box K1 | 15.991 | 80.285 | 0.199 |
| EPHX1 | epoxide hydrolase 1, microsomal (xenobiotic) | 19.966 | 80.059 | 0.249 |
| LOC100507412 | uncharacterized LOC100507412 | 16.224 | 76.580 | 0.212 |
| PNISR | PNN-interacting serine/arginine-rich protein | 14.667 | 68.199 | 0.215 |
| KLF6 | Kruppel-like factor 6 | 16.870 | 67.533 | 0.250 |
| NAT8B | N-acetyltransferase 8B (GCN5-related, putative, gene/pseudogene) | 11.435 | 66.916 | 0.171 |
| ENPP3 | ectonucleotide pyrophosphatase/phosphodiesterase 3 | 13.796 | 64.617 | 0.213 |
| CES3 | carboxylesterase 3 | 15.677 | 62.938 | 0.249 |
| UGT2A3 | UDP glucuronosyltransferase 2 family, polypeptide A3 | 14.366 | 55.769 | 0.258 |
| ADH6 | alcohol dehydrogenase 6 (class V) | 10.375 | 47.515 | 0.218 |
| SLC4A7 | solute carrier family 4, sodium bicarbonate cotransporter, member 7 | 5.345 | 46.316 | 0.115 |
| PRRC2C | proline-rich coiled-coil 2C | 9.762 | 45.819 | 0.213 |
| PPIG | peptidylprolyl isomerase G (cyclophilin G) | 8.958 | 45.269 | 0.198 |
| BRIP1 | BRCA1 interacting protein C-terminal helicase 1 | 7.830 | 42.277 | 0.185 |
| ZBTB7A | zinc finger and BTB domain containing 7A | 9.257 | 40.905 | 0.226 |
| CYP2C19 | cytochrome P450, family 2, subfamily C, polypeptide 19 | 9.722 | 38.122 | 0.255 |
| SCTR | secretin receptor | 9.263 | 37.608 | 0.246 |
| SRRM1 | serine/arginine repetitive matrix 1 | 9.096 | 37.039 | 0.246 |
| CMIP | c-Maf inducing protein | 9.044 | 36.782 | 0.246 |
| MBOAT1 | membrane bound O-acyltransferase domain containing 1 | 8.942 | 36.224 | 0.247 |
| SORD | sorbitol dehydrogenase | 9.207 | 36.200 | 0.254 |
| NT5E | 5'-nucleotidase, ecto (CD73) | 5.385 | 32.860 | 0.164 |
| BRD4 | bromodomain containing 4 | 8.190 | 32.301 | 0.254 |
| UGT1A10 | UDP glucuronosyltransferase 1 family, polypeptide A10 | 4.191 | 31.598 | 0.133 |
| GOLIM4 | golgi integral membrane protein 4 | 6.612 | 29.347 | 0.225 |
| PGM5P2 | phosphoglucomutase 5 pseudogene 2 | 5.042 | 28.849 | 0.175 |
| ANKRD12 | ankyrin repeat domain 12 | 3.441 | 28.545 | 0.121 |
| LDHD | lactate dehydrogenase D | 6.684 | 27.542 | 0.243 |
| FAM133B | family with sequence similarity 133, member B | 3.971 | 26.814 | 0.148 |
| ZNF585B | zinc finger protein 585B | 2.840 | 26.056 | 0.109 |
| SREK1 | splicing regulatory glutamine/lysine-rich protein 1 | 5.854 | 25.981 | 0.225 |
| APOBEC3C | apolipoprotein B mRNA editing enzyme, catalytic polypeptide-like 3C | 6.231 | 25.648 | 0.243 |
| RNA45S5 | RNA, 45S pre-ribosomal 5 | 0.217 | 25.327 | 0.009 |
| CHAD | chondroadherin | 3.300 | 24.923 | 0.132 |
| KMT2E | myeloid/lymphoid or mixed-lineage leukemia 5 (trithorax homolog, Drosophila) | 4.616 | 24.767 | 0.186 |
| SEMA6C | sema domain, transmembrane domain (TM), and cytoplasmic domain, (semaphorin) 6C | 5.729 | 24.009 | 0.239 |
| MATN2 | matrilin 2 | 5.509 | 21.337 | 0.258 |
| PPP1R12A | protein phosphatase 1, regulatory subunit 12A | 4.214 | 21.034 | 0.200 |
| NEMF | nuclear export mediator factor | 3.852 | 20.716 | 0.186 |
| THOC2 | THO complex 2 | 4.449 | 19.894 | 0.224 |
| HMGN5 | high mobility group nucleosome binding domain 5 | 1.530 | 19.839 | 0.077 |
| LOC100507537 | uncharacterized LOC100507537 | 2.564 | 18.811 | 0.136 |
| MAB21L3 | mab-21-like 3 (C. elegans) | 3.713 | 18.235 | 0.204 |
| INO80B | INO80 complex subunit B | 1.505 | 17.544 | 0.086 |
| MSLN | mesothelin | 3.404 | 17.359 | 0.196 |
| RPL19P12 | ribosomal protein L19 pseudogene 12 | 2.086 | 16.938 | 0.123 |
| CYP1A1 | cytochrome P450, family 1, subfamily A, polypeptide 1 | 1.477 | 16.810 | 0.088 |
| ONECUT2 | one cut homeobox 2 | 3.495 | 16.512 | 0.212 |
| SPON1 | spondin 1, extracellular matrix protein | 3.865 | 15.568 | 0.248 |
| PURA | purine-rich element binding protein A | 3.482 | 14.527 | 0.240 |
| TAF3 | TAF3 RNA polymerase II, TATA box binding protein (TBP)-associated factor, 140kDa | 1.361 | 13.509 | 0.101 |
| RSBN1L | round spermatid basic protein 1-like | 1.881 | 12.574 | 0.150 |
| TRIM56 | tripartite motif containing 56 | 3.019 | 12.237 | 0.247 |
| CWF19L2 | CWF19-like 2, cell cycle control (S. pombe) | 2.519 | 12.227 | 0.206 |
| LOC441081 | POM121 membrane glycoprotein (rat) pseudogene | 2.804 | 12.211 | 0.230 |
| CPNE6 | copine VI (neuronal) | 2.135 | 12.155 | 0.176 |
| HSPB6 | heat shock protein, alpha-crystallin-related, B6 | 2.705 | 12.038 | 0.225 |
| HYPK | huntingtin interacting protein K | 1.780 | 11.677 | 0.152 |
| AGAP11 | ankyrin repeat and GTPase domain Arf GTPase activating protein 11 | 2.755 | 11.405 | 0.242 |
| HNRNPH2 | heterogeneous nuclear ribonucleoprotein H2 (H') | 2.166 | 11.191 | 0.194 |
| TLR3 | toll-like receptor 3 | 2.621 | 10.635 | 0.246 |
| SLC34A2 | solute carrier family 34 (sodium phosphate), member 2 | 2.391 | 10.597 | 0.226 |
| SCLY | selenocysteine lyase | 2.307 | 10.590 | 0.218 |
| GCNT4 | glucosaminyl (N-acetyl) transferase 4, core 2 | 1.320 | 10.208 | 0.129 |
| ZNF827 | zinc finger protein 827 | 0.807 | 10.122 | 0.080 |
| ARID4A | AT rich interactive domain 4A (RBP1-like) | 1.578 | 9.851 | 0.160 |
| BOD1L1 | biorientation of chromosomes in cell division 1-like 1 | 1.546 | 9.246 | 0.167 |
| KIAA2026 | KIAA2026 | 1.625 | 9.095 | 0.179 |
| MIA2 | melanoma inhibitory activity 2 | 2.190 | 8.912 | 0.246 |
| CHD7 | chromodomain helicase DNA binding protein 7 | 1.367 | 8.881 | 0.154 |
| ZC3H13 | zinc finger CCCH-type containing 13 | 1.816 | 8.733 | 0.208 |
| PSORS1C3 | psoriasis susceptibility 1 candidate 3 (non-protein coding) | 2.149 | 8.637 | 0.249 |
| SLC5A4 | solute carrier family 5 (low affinity glucose cotransporter), member 4 | 1.529 | 7.993 | 0.191 |
| NEXN | nexilin (F actin binding protein) | 1.037 | 7.890 | 0.131 |
| PHIP | pleckstrin homology domain interacting protein | 1.723 | 7.677 | 0.224 |
| NFIX | nuclear factor I/X (CCAAT-binding transcription factor) | 1.584 | 7.388 | 0.214 |
| BROX | BRO1 domain and CAAX motif containing | 1.802 | 7.383 | 0.244 |
| ESF1 | ESF1, nucleolar pre-rRNA processing protein, homolog (S. cerevisiae) | 1.238 | 7.378 | 0.168 |
| RSF1 | remodeling and spacing factor 1 | 1.802 | 7.244 | 0.249 |
| UGT1A5 | UDP glucuronosyltransferase 1 family, polypeptide A5 | 0.634 | 7.212 | 0.088 |
| PCDP1 | primary ciliary dyskinesia protein 1 | 1.628 | 6.900 | 0.236 |
| SLC5A12 | solute carrier family 5 (sodium/glucose cotransporter), member 12 | 1.470 | 6.872 | 0.214 |
| UGT1A4 | UDP glucuronosyltransferase 1 family, polypeptide A4 | 0.636 | 6.660 | 0.096 |
| LIPF | lipase, gastric | 1.293 | 6.377 | 0.203 |
| CSPP1 | centrosome and spindle pole associated protein 1 | 1.408 | 6.355 | 0.222 |
| PTPLB | protein tyrosine phosphatase-like (proline instead of catalytic arginine), member b | 1.193 | 6.241 | 0.191 |
| LINC01004 | uncharacterized LOC100216546 | 1.303 | 5.948 | 0.219 |
| NBPF10 | neuroblastoma breakpoint family, member 10 | 0.689 | 5.743 | 0.120 |
| ATRX | alpha thalassemia/mental retardation syndrome X-linked | 1.232 | 5.407 | 0.228 |
| UGT1A3 | UDP glucuronosyltransferase 1 family, polypeptide A3 | 0.806 | 4.890 | 0.165 |
| KIAA0825 | KIAA0825 | 0.800 | 4.752 | 0.168 |
| H2AFB3 | H2A histone family, member B3 | 0.434 | 4.542 | 0.096 |
| SCARNA9 | small Cajal body-specific RNA 9 | 1.077 | 4.514 | 0.238 |
| AMPD1 | adenosine monophosphate deaminase 1 | 0.785 | 4.333 | 0.181 |
| KCNJ13 | potassium inwardly-rectifying channel, subfamily J, member 13 | 0.860 | 4.161 | 0.207 |
| FAM133DP | family with sequence similarity 133, member A pseudogene | 0.998 | 4.043 | 0.247 |
| ARHGEF34P | Rho guanine nucleotide exchange factor (GEF) 5 pseudogene | 1.006 | 3.962 | 0.254 |
| SEMA3E | sema domain, immunoglobulin domain (Ig), short basic domain, secreted, (semaphorin) 3E | 0.765 | 3.935 | 0.194 |
| LOC202181 | SUMO-interacting motifs containing 1 pseudogene | 0.685 | 3.720 | 0.184 |
| SLC6A12 | solute carrier family 6 (neurotransmitter transporter, betaine/GABA), member 12 | 0.603 | 3.693 | 0.163 |
| ACRBP | acrosin binding protein | 0.939 | 3.650 | 0.257 |
| RPL21P28 | ribosomal protein L21 pseudogene 28 | 0.893 | 3.643 | 0.245 |
| DPRXP4 | divergent-paired related homeobox pseudogene 4 | 0.188 | 3.503 | 0.054 |
| ZBTB20 | zinc finger and BTB domain containing 20 | 0.377 | 3.441 | 0.110 |
| TBC1D3 | TBC1 domain family, member 3 | 0.236 | 3.422 | 0.069 |
| EBLN2 | endogenous Bornavirus-like nucleoprotein 2 | 0.507 | 3.405 | 0.149 |
| CNTFR | ciliary neurotrophic factor receptor | 0.595 | 3.269 | 0.182 |
| MTVR2 | mouse mammary tumor virus receptor homolog 2 | 0.623 | 3.085 | 0.202 |
| PIPSL | PIP5K1A and PSMD4-like, pseudogene | 0.067 | 3.024 | 0.022 |
| NCOA2 | nuclear receptor coactivator 2 | 0.703 | 3.014 | 0.233 |
| HRH2 | histamine receptor H2 | 0.635 | 2.986 | 0.213 |
| TRHDE | thyrotropin-releasing hormone degrading enzyme | 0.733 | 2.966 | 0.247 |
| ZNF281 | zinc finger protein 281 | 0.643 | 2.824 | 0.228 |
| ANKRD36 | ankyrin repeat domain 36 | 0.261 | 2.780 | 0.094 |
| FAM222A | family with sequence similarity 222, member A | 0.606 | 2.769 | 0.219 |
| SIGLEC15 | sialic acid binding Ig-like lectin 15 | 0.275 | 2.665 | 0.103 |
| KCNK15 | potassium channel, subfamily K, member 15 | 0.572 | 2.647 | 0.216 |
| MRPL42P5 | mitochondrial ribosomal protein L42 pseudogene 5 | 0.535 | 2.584 | 0.207 |
| LOC93432 | maltase-glucoamylase (alpha-glucosidase) pseudogene | 0.588 | 2.510 | 0.234 |
| HERC2P3 | hect domain and RLD 2 pseudogene 3 | 0.489 | 2.445 | 0.200 |
| ACPT | acid phosphatase, testicular | 0.079 | 2.430 | 0.033 |
| TMEM132E | transmembrane protein 132E | 0.509 | 2.399 | 0.212 |
| CNDP1 | carnosine dipeptidase 1 (metallopeptidase M20 family) | 0.415 | 2.367 | 0.175 |
| TLX1 | T-cell leukemia homeobox 1 | 0.515 | 2.353 | 0.219 |
| MS4A15 | membrane-spanning 4-domains, subfamily A, member 15 | 0.393 | 2.303 | 0.171 |
| SUMO1P3 | SUMO1 pseudogene 3 | 0.384 | 2.299 | 0.167 |
| UGT1A7 | UDP glucuronosyltransferase 1 family, polypeptide A7 | 0.269 | 2.249 | 0.119 |
| RAD54L2 | RAD54-like 2 (S. cerevisiae) | 0.378 | 2.176 | 0.174 |
| MAP1B | microtubule-associated protein 1B | 0.251 | 2.154 | 0.117 |
| ANKRD20A4 | ankyrin repeat domain 20 family, member A4 | 0.004 | 2.099 | 0.002 |
| DIRAS2 | DIRAS family, GTP-binding RAS-like 2 | 0.421 | 2.066 | 0.204 |
| OR2A4 | olfactory receptor, family 2, subfamily A, member 4 | 0.430 | 2.049 | 0.210 |
| ANKRD26 | ankyrin repeat domain 26 | 0.463 | 1.997 | 0.232 |
| SFRP2 | secreted frizzled-related protein 2 | 0.428 | 1.928 | 0.222 |
| LMBRD2 | LMBR1 domain containing 2 | 0.490 | 1.919 | 0.255 |
| FAM25A | family with sequence similarity 25, member A | 0.229 | 1.840 | 0.124 |
| FAM74A3 | family with sequence similarity 74, member A3 | 0.354 | 1.707 | 0.207 |
| NUTM2D | NUT family member 2D | 0.288 | 1.514 | 0.190 |
| TPTE2P6 | transmembrane phosphoinositide 3-phosphatase and tensin homolog 2 pseudogene 6 | 0.107 | 1.367 | 0.078 |
| ANKRD20A1 | ankyrin repeat domain 20 family, member A1 | 0.102 | 1.367 | 0.075 |
| GOLGA6L6 | golgin A6 family-like 6 | 0.336 | 1.338 | 0.251 |
| COL25A1 | collagen, type XXV, alpha 1 | 0.095 | 1.296 | 0.073 |
| HLF | hepatic leukemia factor | 0.290 | 1.284 | 0.226 |
| TACR2 | tachykinin receptor 2 | 0.324 | 1.265 | 0.256 |
| ANKRD20A11P | ankyrin repeat domain 20 family, member A11, pseudogene | 0.006 | 1.256 | 0.005 |
| HOXA7 | homeobox A7 | 0.063 | 1.249 | 0.050 |
| SATB2 | SATB homeobox 2 | 0.281 | 1.227 | 0.229 |
| BDNF | brain-derived neurotrophic factor | 0.102 | 1.184 | 0.086 |
| SND1-IT1 | SND1 intronic transcript 1 (non-protein coding) | 0.117 | 1.142 | 0.102 |
| TGIF2-C20orf24 | TGIF2-C20orf24 readthrough | 0.028 | 1.117 | 0.025 |
| CYP3A7 | cytochrome P450, family 3, subfamily A, polypeptide 7 | 0.250 | 1.104 | 0.226 |
| LYPD8 | LY6/PLAUR domain containing 8 | 0.149 | 1.096 | 0.136 |
| FLJ42393 | uncharacterized LOC401105 | 0.087 | 1.095 | 0.080 |
| NEK10 | NIMA-related kinase 10 | 0.132 | 1.057 | 0.125 |
| ANKRD20A5P | ankyrin repeat domain 20 family, member A5, pseudogene | 0.164 | 1.022 | 0.160 |

**Table S6 DEGs increased in children with SAM compared to children with stunting**

| genename | description | meanfpkm_sam | meanfpkm_stunted | ratiosamstunted |
| --- | --- | --- | --- | --- |
| MTRNR2L8 | MT-RNR2-like 8 | 26460.5410 | 2464.3904 | 10.7372 |
| MTRNR2L1 | MT-RNR2-like 1 | 13362.7256 | 375.5569 | 35.5811 |
| MIR650 | microRNA 650 | 3699.5244 | 3.9895 | 927.3173 |
| MTRNR2L10 | MT-RNR2-like 10 | 2828.8354 | 81.2495 | 34.8167 |
| MTRNR2L6 | MT-RNR2-like 6 | 2762.7886 | 129.4386 | 21.3444 |
| MT1G | metallothionein 1G | 2080.7068 | 145.2641 | 14.3236 |
| MT2A | metallothionein 2A | 1872.6636 | 243.8878 | 7.6784 |
| MTRNR2L4 | MT-RNR2-like 4 | 1784.7836 | 35.8071 | 49.8444 |
| MTRNR2L3 | MT-RNR2-like 3 | 1412.6686 | 14.8025 | 95.4342 |
| PGC | progastricsin (pepsinogen C) | 983.6212 | 42.0017 | 23.4186 |
| MT1H | metallothionein 1H | 907.1454 | 27.1125 | 33.4585 |
| MT1E | metallothionein 1E | 680.6407 | 63.0010 | 10.8036 |
| ELK2AP | ELK2A, member of ETS oncogene family, pseudogene | 483.3057 | 76.0514 | 6.3550 |
| IGLL3P | immunoglobulin lambda-like polypeptide 3, pseudogene | 464.7431 | 4.8836 | 95.1648 |
| TFF2 | trefoil factor 2 | 462.2443 | 60.0375 | 7.6993 |
| MT1X | metallothionein 1X | 403.8345 | 27.8161 | 14.5180 |
| MT1F | metallothionein 1F | 336.0009 | 26.4492 | 12.7037 |
| TFF1 | trefoil factor 1 | 323.0379 | 50.0632 | 6.4526 |
| MT1M | metallothionein 1M | 293.5200 | 4.9158 | 59.7100 |
| IGLL1 | immunoglobulin lambda-like polypeptide 1 | 275.8381 | 18.3941 | 14.9960 |
| SAA1 | serum amyloid A1 | 248.7888 | 24.6827 | 10.0795 |
| MT1B | metallothionein 1B | 222.1893 | 1.2114 | 183.4220 |
| BMS1P20 | BMS1 homolog, ribosome assembly protein (yeast) pseudogene | 212.1443 | 22.8708 | 9.2758 |
| S100P | S100 calcium binding protein P | 209.8814 | 10.7881 | 19.4548 |
| MINOS1-NBL1 | C1orf151-NBL1 readthrough | 156.2507 | 0.9620 | 162.4171 |
| KIAA0101 | KIAA0101 | 146.4269 | 11.9729 | 12.2299 |
| ATP1A1-AS1 | ATP1A1 opposite strand | 135.2245 | 2.3875 | 56.6395 |
| C6orf58 | chromosome 6 open reading frame 58 | 123.0290 | 8.9993 | 13.6709 |
| PI3 | peptidase inhibitor 3, skin-derived | 122.9114 | 5.1895 | 23.6847 |
| CBR3-AS1 | CBR3 antisense RNA 1 | 122.5621 | 3.7678 | 32.5289 |
| MUC1 | mucin 1, cell surface associated | 121.0155 | 17.4727 | 6.9260 |
| CCDC152 | coiled-coil domain containing 152 | 119.5493 | 2.7319 | 43.7611 |
| ANKRD19P | ankyrin repeat domain 19, pseudogene | 107.4957 | 1.1968 | 89.8208 |
| SNX22 | sorting nexin 22 | 104.3559 | 7.0347 | 14.8344 |
| PSCA | prostate stem cell antigen | 102.4343 | 0.4444 | 230.4967 |
| GGTLC2 | gamma-glutamyltransferase light chain 2 | 102.3143 | 1.5946 | 64.1639 |
| FTX | FTX transcript, XIST regulator (non-protein coding) | 90.9017 | 2.2741 | 39.9732 |
| LOC401242 | uncharacterized LOC401242 | 87.0162 | 0.2375 | 366.4493 |
| LOC100506385 | uncharacterized LOC100506385 | 80.4881 | 7.9902 | 10.0734 |
| LOC100133286 | uncharacterized LOC100133286 | 76.7386 | 0.9825 | 78.1020 |
| KDM4A-AS1 | KDM4A antisense RNA 1 | 65.3998 | 5.5341 | 11.8177 |
| DDTL | D-dopachrome tautomerase-like | 62.5064 | 6.4871 | 9.6355 |
| SARM1 | sterile alpha and TIR motif containing 1 | 56.5705 | 2.4227 | 23.3501 |
| LOC284889 | uncharacterized LOC284889 | 55.7276 | 1.5437 | 36.0994 |
| POLR3H | polymerase (RNA) III (DNA directed) polypeptide H (22.9kD) | 55.5798 | 4.8893 | 11.3676 |
| GAS6-AS2 | uncharacterized LOC100506394 | 54.2521 | 6.5663 | 8.2622 |
| SNHG4 | small nucleolar RNA host gene 4 (non-protein coding) | 53.6745 | 6.4946 | 8.2645 |
| DYDC1 | DPY30 domain containing 1 | 53.1086 | 2.6983 | 19.6822 |
| LINC00987 | uncharacterized LOC100499405 | 52.9936 | 1.4275 | 37.1244 |
| NME7 | NME/NM23 family member 7 | 50.6786 | 1.8725 | 27.0640 |
| MAN1B1-AS1 | uncharacterized LOC100289341 | 48.8236 | 2.6831 | 18.1970 |
| GPR82 | G protein-coupled receptor 82 | 47.7650 | 6.6592 | 7.1728 |
| TAPT1-AS1 | TAPT1 antisense RNA 1 (head to head) | 46.5679 | 1.4759 | 31.5515 |
| LINC01138 | uncharacterized FLJ39739 | 44.6945 | 3.5705 | 12.5177 |
| SLPI | secretory leukocyte peptidase inhibitor | 42.0083 | 4.5627 | 9.2069 |
| FAM166A | family with sequence similarity 166, member A | 41.7412 | 0.8312 | 50.2188 |
| FAM74A1 | family with sequence similarity 74, member A1 | 40.9969 | 6.5156 | 6.2921 |
| CC2D2B | coiled-coil and C2 domain containing 2B | 38.3514 | 0.1636 | 234.4802 |
| MT1L | metallothionein 1L (gene/pseudogene) | 38.2279 | 1.9461 | 19.6433 |
| MIR1273A | microRNA 1273a | 38.1574 | 1.9308 | 19.7620 |
| CSTF3-AS1 | CSTF3 antisense RNA 1 (head to head) | 37.8174 | 0.7134 | 53.0108 |
| OLAH | oleoyl-ACP hydrolase | 37.5000 | 2.8041 | 13.3734 |
| C5orf45 | chromosome 5 open reading frame 45 | 37.4964 | 1.9649 | 19.0830 |
| LINC01012 | uncharacterized LOC100507173 | 37.3707 | 3.9624 | 9.4314 |
| LEPR | leptin receptor | 36.8814 | 2.6832 | 13.7452 |
| ZNF818P | zinc finger protein 818, pseudogene | 36.7171 | 5.9456 | 6.1755 |
| LOC100652768 | uncharacterized LOC100652768 | 36.6138 | 4.8990 | 7.4738 |
| CAPN12 | calpain 12 | 34.6462 | 5.0276 | 6.8912 |
| RUSC1-AS1 | RUSC1 antisense RNA 1 | 33.9417 | 1.1346 | 29.9157 |
| FOXH1 | forkhead box H1 | 33.8012 | 0.8376 | 40.3535 |
| LINC01021 | uncharacterized LOC643401 | 33.7988 | 1.3893 | 24.3276 |
| MIR5047 | microRNA 5047 | 33.6926 | 1.3990 | 24.0837 |
| LOC349160 | uncharacterized LOC349160 | 32.8712 | 0.0788 | 417.0753 |
| C2orf15 | chromosome 2 open reading frame 15 | 31.0955 | 1.4410 | 21.5788 |
| BLOC1S1-RDH5 | BLOC1S1-RDH5 readthrough | 30.9848 | 4.5259 | 6.8461 |
| LL22NC01-81G9.3 | uncharacterized protein FLJ39582-like | 30.4112 | 1.0151 | 29.9593 |
| DUOXA1 | dual oxidase maturation factor 1 | 29.9914 | 0.5298 | 56.6057 |
| GKN1 | gastrokine 1 | 29.6314 | 0.0032 | 9201.3379 |
| MT1A | metallothionein 1A | 29.2790 | 0.8749 | 33.4650 |
| SNORA70 | small nucleolar RNA, H/ACA box 70 | 29.1817 | 1.5419 | 18.9262 |
| ATP6V1C2 | ATPase, H+ transporting, lysosomal 42kDa, V1 subunit C2 | 27.7605 | 0.6920 | 40.1143 |
| RHBG | Rh family, B glycoprotein (gene/pseudogene) | 26.8348 | 3.9075 | 6.8676 |
| PSMC3IP | PSMC3 interacting protein | 25.6374 | 2.5398 | 10.0941 |
| CATSPER2P1 | cation channel, sperm associated 2 pseudogene 1 | 25.5760 | 2.2415 | 11.4101 |
| LOC101243545 | uncharacterized LOC101243545 | 25.2021 | 0.1968 | 128.0729 |
| C14orf178 | chromosome 14 open reading frame 178 | 24.9919 | 1.2846 | 19.4554 |
| AARD | alanine and arginine rich domain containing protein | 24.8243 | 1.1073 | 22.4190 |
| KCTD21-AS1 | uncharacterized LOC100289388 | 24.1157 | 0.3103 | 77.7077 |
| FAM151A | family with sequence similarity 151, member A | 24.0083 | 1.3502 | 17.7817 |
| LOC283731 | uncharacterized LOC283731 | 21.5162 | 2.3754 | 9.0578 |
| BMP6 | bone morphogenetic protein 6 | 21.1014 | 0.5853 | 36.0551 |
| APOC1P1 | apolipoprotein C-I pseudogene 1 | 20.2183 | 1.4981 | 13.4957 |
| FAIM | Fas apoptotic inhibitory molecule | 20.1819 | 3.0422 | 6.6340 |
| NME1-NME2 | NME1-NME2 readthrough | 20.0557 | 2.2568 | 8.8869 |
| THAP7-AS1 | THAP7 antisense RNA 1 | 19.9940 | 1.5373 | 13.0061 |
| PHKG1 | phosphorylase kinase, gamma 1 (muscle) | 19.6429 | 1.6351 | 12.0134 |
| PCDHB9 | protocadherin beta 9 | 19.0145 | 1.8285 | 10.3991 |
| LINC00907 | uncharacterized LOC284260 | 18.9438 | 1.5920 | 11.8991 |
| EMILIN3 | elastin microfibril interfacer 3 | 18.7355 | 2.9158 | 6.4256 |
| BAIAP2-AS1 | BAIAP2 antisense RNA 1 (head to head) | 18.6248 | 1.9442 | 9.5795 |
| LINC00485 | long intergenic non-protein coding RNA 485 | 18.5621 | 2.2790 | 8.1449 |
| ATP5J2-PTCD1 | ATP5J2-PTCD1 readthrough | 18.1914 | 0.9459 | 19.2312 |
| LOC100128531 | uncharacterized LOC100128531 | 17.7586 | 2.4686 | 7.1937 |
| ZNF283 | zinc finger protein 283 | 17.5543 | 1.6234 | 10.8134 |
| LINC00330 | long intergenic non-protein coding RNA 330 | 17.4212 | 2.1792 | 7.9945 |
| ZACN | zinc activated ligand-gated ion channel | 17.3981 | 0.0937 | 185.6216 |
| LINC00381 | long intergenic non-protein coding RNA 381 | 17.1464 | 1.2946 | 13.2448 |
| NANOGNB | NANOG neighbor homeobox | 16.4543 | 0.2290 | 71.8581 |
| MIR497HG | mir-497-195 cluster host gene (non-protein coding) | 16.3555 | 2.4046 | 6.8018 |
| C6orf25 | chromosome 6 open reading frame 25 | 16.0098 | 1.5776 | 10.1480 |
| CEACAM7 | carcinoembryonic antigen-related cell adhesion molecule 7 | 15.9743 | 0.8553 | 18.6778 |
| GKN2 | gastrokine 2 | 15.6976 | 0.0534 | 294.0189 |
| A2M-AS1 | A2M antisense RNA 1 | 15.3555 | 0.3266 | 47.0147 |
| SKP1P2 | S-phase kinase-associated protein 1 pseudogene 2 | 15.2190 | 0.9222 | 16.5029 |
| RPL17-C18orf32 | RPL17-C18orf32 readthrough | 15.1038 | 0.6646 | 22.7270 |
| NRIP2 | nuclear receptor interacting protein 2 | 14.9664 | 1.9014 | 7.8714 |
| ZNF32-AS1 | ZNF32 antisense RNA 1 | 14.7419 | 0.0581 | 253.5780 |
| TMEM183B | transmembrane protein 183B | 14.0474 | 2.0741 | 6.7729 |
| LOC100128573 | uncharacterized LOC100128573 | 13.5983 | 0.8663 | 15.6975 |
| SNORA31 | small nucleolar RNA, H/ACA box 31 | 13.5864 | 0.2569 | 52.8759 |
| ASAH2B | N-acylsphingosine amidohydrolase (non-lysosomal ceramidase) 2B | 13.5812 | 1.2054 | 11.2667 |
| ZFP30 | ZFP30 zinc finger protein | 13.5405 | 1.6136 | 8.3917 |
| CLDN18 | claudin 18 | 13.5212 | 0.4880 | 27.7093 |
| PCNA-AS1 | PCNA antisense RNA 1 | 13.4864 | 0.7707 | 17.4994 |
| MT1DP | metallothionein 1D, pseudogene | 13.4226 | 0.5942 | 22.5880 |
| SNORA61 | small nucleolar RNA, H/ACA box 61 | 13.1757 | 0.2000 | 65.8786 |
| SLX1B-SULT1A4 | SLX1B-SULT1A4 readthrough | 13.0705 | 0.0132 | 988.6642 |
| SHANK2-AS3 | SHANK2 antisense RNA 3 | 12.6021 | 0.6646 | 18.9627 |
| PRR7-AS1 | PRR7 antisense RNA 1 | 12.5619 | 1.9342 | 6.4945 |
| RNY3 | RNA, Ro-associated Y3 | 12.4883 | 1.0249 | 12.1847 |
| ZNF670-ZNF695 | ZNF670-ZNF695 readthrough | 12.3740 | 1.8156 | 6.8154 |
| SNORA67 | small nucleolar RNA, H/ACA box 67 | 12.2800 | 1.8069 | 6.7960 |
| TMEM254-AS1 | TMEM254 antisense RNA 1 | 12.0381 | 0.8725 | 13.7966 |
| MED4-AS1 | MED4 antisense RNA 1 | 12.0136 | 0.2375 | 50.5925 |
| ZNF542P | zinc finger protein 542 | 11.8800 | 1.8356 | 6.4720 |
| CCDC42B | coiled-coil domain containing 42B | 11.6040 | 0.8236 | 14.0901 |
| BSN-AS2 | BSN antisense RNA 2 (head to head) | 11.3800 | 0.7712 | 14.7565 |
| LOC646736 | uncharacterized LOC646736 | 11.0631 | 0.1354 | 81.6924 |
| PRSS1 | protease, serine, 1 (trypsin 1) | 11.0210 | 0.2553 | 43.1764 |
| PPAN-P2RY11 | PPAN-P2RY11 readthrough | 11.0000 | 0.1556 | 70.6972 |
| CNTD1 | cyclin N-terminal domain containing 1 | 10.6550 | 1.1186 | 9.5249 |
| MAP1LC3B2 | microtubule-associated protein 1 light chain 3 beta 2 | 10.6114 | 1.6120 | 6.5826 |
| DPY19L1P1 | dpy-19-like 1 pseudogene 1 (C. elegans) | 10.5962 | 0.8761 | 12.0947 |
| HIF1A-AS2 | HIF1A antisense RNA 2 | 10.4895 | 0.1381 | 75.9364 |
| CYP21A2 | cytochrome P450, family 21, subfamily A, polypeptide 2 | 10.4393 | 0.6573 | 15.8824 |
| TNXA | tenascin XA (pseudogene) | 10.3590 | 0.8312 | 12.4630 |
| IL31RA | interleukin 31 receptor A | 10.3048 | 0.3142 | 32.7929 |
| PMCHL2 | pro-melanin-concentrating hormone-like 2, pseudogene | 10.2979 | 0.3792 | 27.1602 |
| FIRRE | family with sequence similarity 195, member A pseudogene | 10.2819 | 1.3710 | 7.4995 |
| PRR4 | proline rich 4 (lacrimal) | 10.1438 | 0.8119 | 12.4945 |
| HCG25 | HLA complex group 25 (non-protein coding) | 10.0386 | 0.5034 | 19.9419 |
| LRRC2 | leucine rich repeat containing 2 | 10.0021 | 1.3515 | 7.4006 |
| HCG4 | HLA complex group 4 (non-protein coding) | 9.8617 | 1.0897 | 9.0502 |
| RFPL1S | RFPL1 antisense RNA 1 | 9.8167 | 1.0214 | 9.6114 |
| PIN4P1 | protein (peptidylprolyl cis/trans isomerase) NIMA-interacting, 4 pseudogene 1 | 9.7440 | 0.7227 | 13.4826 |
| EIF3CL | eukaryotic translation initiation factor 3, subunit C-like | 9.6588 | 1.1734 | 8.2315 |
| FAM41C | family with sequence similarity 41, member C | 9.5381 | 0.7722 | 12.3518 |
| EME1 | essential meiotic endonuclease 1 homolog 1 (S. pombe) | 9.0621 | 0.7180 | 12.6220 |
| LSMEM2 | leucine-rich single-pass membrane protein 2 | 9.0155 | 0.2480 | 36.3577 |
| LOC100129148 | uncharacterized LOC100129148 | 8.8890 | 0.0619 | 143.6860 |
| LINC00114 | long intergenic non-protein coding RNA 114 | 8.8240 | 0.2856 | 30.8973 |
| LOC100287042 | uncharacterized LOC100287042 | 8.8205 | 0.9246 | 9.5400 |
| CNGB1 | cyclic nucleotide gated channel beta 1 | 8.7898 | 0.8714 | 10.0875 |
| LOC400794 | uncharacterized LOC400794 | 8.7779 | 0.5129 | 17.1148 |
| CXCL17 | chemokine (C-X-C motif) ligand 17 | 8.7407 | 0.3986 | 21.9261 |
| SCARNA22 | small Cajal body-specific RNA 22 | 8.6064 | 0.6286 | 13.6905 |
| SHISA2 | shisa homolog 2 (Xenopus laevis) | 8.5419 | 1.0842 | 7.8783 |
| GCSAML | germinal center-associated, signaling and motility-like | 8.5267 | 0.1273 | 66.9871 |
| EP400NL | EP400 N-terminal like | 8.4898 | 0.6968 | 12.1843 |
| RAB4B-EGLN2 | RAB4B-EGLN2 readthrough (non-protein coding) | 8.4574 | 0.3705 | 22.8264 |
| LOC100129931 | uncharacterized LOC100129931 | 8.4305 | 1.3415 | 6.2842 |
| LOC100131626 | uncharacterized LOC100131626 | 8.4124 | 0.5751 | 14.6281 |
| LOC400548 | uncharacterized LOC400548 | 8.2857 | 0.7386 | 11.2175 |
| PDZD9 | PDZ domain containing 9 | 8.2733 | 0.1329 | 62.2611 |
| S100A8 | S100 calcium binding protein A8 | 8.2462 | 1.1625 | 7.0932 |
| CXorf31 | chromosome X open reading frame 31 | 8.1076 | 0.0908 | 89.2443 |
| LOC283335 | uncharacterized LOC283335 | 8.0929 | 1.0649 | 7.5995 |
| LINC00598 | long intergenic non-protein coding RNA 598 | 7.8490 | 0.9278 | 8.4599 |
| MIR4516 | microRNA 4516 | 7.8198 | 0.8403 | 9.3055 |
| LOC100507462 | uncharacterized LOC100507462 | 7.7238 | 0.2786 | 27.7193 |
| MIA-RAB4B | MIA-RAB4B readthrough | 7.6419 | 0.5095 | 14.9991 |
| PGA4 | pepsinogen 4, group I (pepsinogen A) | 7.3755 | 0.1007 | 73.2581 |
| F11-AS1 | uncharacterized LOC285441 | 7.1755 | 0.5114 | 14.0323 |
| LOC100379224 | uncharacterized LOC100379224 | 7.1060 | 0.4341 | 16.3706 |
| SYNC | syncoilin, intermediate filament protein | 7.0779 | 0.7173 | 9.8675 |
| CCDC73 | coiled-coil domain containing 73 | 7.0683 | 0.1122 | 62.9957 |
| FAM106A | family with sequence similarity 106, member A | 7.0664 | 1.1314 | 6.2460 |
| DUSP19 | dual specificity phosphatase 19 | 6.9998 | 0.9817 | 7.1303 |
| CCL16 | chemokine (C-C motif) ligand 16 | 6.8264 | 0.4620 | 14.7747 |
| MT1JP | metallothionein 1J, pseudogene | 6.8126 | 0.1188 | 57.3387 |
| NRSN1 | neurensin 1 | 6.6531 | 0.4581 | 14.5221 |
| NDUFB2-AS1 | NDUFB2 antisense RNA 1 | 6.6226 | 0.8153 | 8.1234 |
| LINC00671 | long intergenic non-protein coding RNA 671 | 6.5652 | 0.7686 | 8.5413 |
| FAM172BP | family with sequence similarity 172, member B pseudogene | 6.4129 | 0.5349 | 11.9885 |
| PRR29 | chromosome 17 open reading frame 72 | 6.2583 | 0.6892 | 9.0812 |
| SAP30L-AS1 | uncharacterized LOC386627 | 6.2067 | 0.6342 | 9.7860 |
| GOLGA6L22 | putative golgin subfamily A member 6-like | 6.1774 | 0.8220 | 7.5148 |
| BEND2 | BEN domain containing 2 | 6.0950 | 0.2844 | 21.4306 |
| CXCL5 | chemokine (C-X-C motif) ligand 5 | 5.9979 | 0.5842 | 10.2661 |
| FBXL13 | F-box and leucine-rich repeat protein 13 | 5.8824 | 0.4205 | 13.9887 |
| TMPRSS11B | transmembrane protease, serine 11B | 5.8302 | 0.0693 | 84.1037 |
| OTX2-AS1 | OTX2 antisense RNA 1 (head to head) | 5.7933 | 0.5322 | 10.8856 |
| UBE2F-SCLY | UBE2F-SCLY readthrough | 5.7862 | 0.2861 | 20.2242 |
| SLC5A10 | solute carrier family 5 (sodium/glucose cotransporter), member 10 | 5.7814 | 0.3342 | 17.2974 |
| MROH7-TTC4 | HEATR8-TTC4 readthrough | 5.7645 | 0.5080 | 11.3482 |
| SLMO2-ATP5E | SLMO2-ATP5E readthrough | 5.7364 | 0.4600 | 12.4705 |
| PBOV1 | prostate and breast cancer overexpressed 1 | 5.6705 | 0.7766 | 7.3016 |
| NEDD8-MDP1 | NEDD8-MDP1 readthrough | 5.6450 | 0.5346 | 10.5598 |
| LINC00883 | uncharacterized LOC344595 | 5.6295 | 0.4907 | 11.4729 |
| GP6 | glycoprotein VI (platelet) | 5.4664 | 0.7864 | 6.9508 |
| TCEB3-AS1 | uncharacterized LOC100506963 | 5.3807 | 0.8180 | 6.5782 |
| SUGT1P3 | suppressor of G2 allele of SKP1 (S. cerevisiae) pseudogene 3 | 5.3645 | 0.5293 | 10.1347 |
| MYL10 | myosin, light chain 10, regulatory | 5.3179 | 0.1164 | 45.6701 |
| FCGR1C | Fc fragment of IgG, high affinity Ic, receptor (CD64), pseudogene | 5.2955 | 0.5239 | 10.1078 |
| RAET1E | retinoic acid early transcript 1E | 5.1890 | 0.5714 | 9.0820 |
| RNY1 | RNA, Ro-associated Y1 | 5.1855 | 0.5022 | 10.3254 |
| RNVU1-7 | RNA, U1 small nuclear 9 | 5.1379 | 0.8324 | 6.1725 |
| SYCP3 | synaptonemal complex protein 3 | 5.0569 | 0.4712 | 10.7323 |
| SNORA57 | small nucleolar RNA, H/ACA box 57 | 4.9760 | 0.7344 | 6.7755 |
| ENTPD3-AS1 | ENTPD3 antisense RNA 1 | 4.9629 | 0.6937 | 7.1539 |
| CHRNE | cholinergic receptor, nicotinic, epsilon (muscle) | 4.9538 | 0.3397 | 14.5846 |
| PLCXD3 | phosphatidylinositol-specific phospholipase C, X domain containing 3 | 4.9195 | 0.1710 | 28.7663 |
| RPS17 | ribosomal protein S17 | 4.8667 | 0.0792 | 61.4847 |
| ANKS1B | ankyrin repeat and sterile alpha motif domain containing 1B | 4.8505 | 0.4793 | 10.1195 |
| DEFA1B | defensin, alpha 1B | 4.8319 | 0.0934 | 51.7391 |
| ISPD | isoprenoid synthase domain containing | 4.8176 | 0.7064 | 6.8196 |
| PAPL | iron/zinc purple acid phosphatase-like protein | 4.7405 | 0.3680 | 12.8829 |
| IRGM | immunity-related GTPase family, M | 4.7088 | 0.7173 | 6.5647 |
| C12orf77 | chromosome 12 open reading frame 77 | 4.7081 | 0.5547 | 8.4869 |
| B3GAT2 | beta-1,3-glucuronyltransferase 2 (glucuronosyltransferase S) | 4.6905 | 0.2695 | 17.4049 |
| CASKIN1 | CASK interacting protein 1 | 4.6764 | 0.0851 | 54.9620 |
| LINC00948 | uncharacterized LOC100507027 | 4.6210 | 0.0492 | 94.0125 |
| EHHADH-AS1 | EHHADH antisense RNA 1 | 4.5714 | 0.0275 | 166.4903 |
| RPS10-NUDT3 | RPS10-NUDT3 readthrough | 4.5340 | 0.0310 | 146.1797 |
| DEFB4A | defensin, beta 4A | 4.4452 | 0.0400 | 111.1310 |
| LINC01010 | uncharacterized LOC154092 | 4.4250 | 0.2927 | 15.1173 |
| EGFLAM-AS4 | EGFLAM antisense RNA 4 | 4.3669 | 0.2864 | 15.2454 |
| IGFBP7-AS1 | uncharacterized LOC255130 | 4.3421 | 0.1222 | 35.5321 |
| OR6W1P | olfactory receptor, family 6, subfamily W, member 1 pseudogene | 4.2795 | 0.5731 | 7.4680 |
| SNORA45B | small nucleolar RNA, H/ACA box 45 | 4.2286 | 0.0368 | 114.9704 |
| SNORD3C | small nucleolar RNA, C/D box 3C | 4.1990 | 0.0615 | 68.2490 |
| SNORA76C | small nucleolar RNA, H/ACA box 76 | 4.1762 | 0.5932 | 7.0399 |
| LOC653712 | intraflagellar transport 122 homolog (Chlamydomonas) pseudogene | 4.1688 | 0.4544 | 9.1742 |
| LINC00331 | long intergenic non-protein coding RNA 331 | 4.1657 | 0.1392 | 29.9363 |
| LOC286190 | uncharacterized LOC286190 | 4.1626 | 0.4714 | 8.8312 |
| MIR570 | microRNA 570 | 4.1464 | 0.1307 | 31.7301 |
| SAA4 | serum amyloid A4, constitutive | 4.0617 | 0.4703 | 8.6356 |
| GTF2IRD1P1 | GTF2I repeat domain containing 1 pseusogene 1 | 4.0569 | 0.0180 | 225.8089 |
| DPCR1 | diffuse panbronchiolitis critical region 1 | 4.0100 | 0.3019 | 13.2841 |
| HLA-DPB2 | major histocompatibility complex, class II, DP beta 2 (pseudogene) | 3.9855 | 0.3398 | 11.7278 |
| TEX101 | testis expressed 101 | 3.9760 | 0.5834 | 6.8153 |
| SSX5 | synovial sarcoma, X breakpoint 5 | 3.9607 | 0.0329 | 120.4547 |
| LINC00466 | long intergenic non-protein coding RNA 466 | 3.8843 | 0.2941 | 13.2088 |
| NXNL2 | nucleoredoxin-like 2 | 3.8631 | 0.5020 | 7.6949 |
| MKNK1-AS1 | MKNK1 antisense RNA 1 | 3.8167 | 0.2125 | 17.9572 |
| COMMD3-BMI1 | COMMD3-BMI1 readthrough | 3.8076 | 0.2097 | 18.1608 |
| PIH1D3 | PIH1 domain containing 3 | 3.7343 | 0.3483 | 10.7213 |
| SERHL | serine hydrolase-like | 3.6843 | 0.1302 | 28.3038 |
| PRCD | progressive rod-cone degeneration | 3.6786 | 0.2071 | 17.7607 |
| LOC729987 | uncharacterized LOC729987 | 3.6448 | 0.5864 | 6.2151 |
| SNORA18 | small nucleolar RNA, H/ACA box 18 | 3.6360 | 0.2037 | 17.8470 |
| SNORA63 | small nucleolar RNA, H/ACA box 63 | 3.6167 | 0.4161 | 8.6918 |
| HPCA | hippocalcin | 3.5943 | 0.2534 | 14.1848 |
| BMS1P17 | ribosome biogenesis protein BMS1 homolog pseudogene | 3.5493 | 0.3371 | 10.5283 |
| SNORA7B | small nucleolar RNA, H/ACA box 7B | 3.5186 | 0.0997 | 35.3054 |
| SERINC4 | serine incorporator 4 | 3.4329 | 0.3268 | 10.5051 |
| C3orf27 | chromosome 3 open reading frame 27 | 3.3714 | 0.4139 | 8.1455 |
| XKR5 | XK, Kell blood group complex subunit-related family, member 5 | 3.3562 | 0.0815 | 41.1674 |
| LOC100131347 | RAD52 motif 1 pseudogene | 3.3157 | 0.2812 | 11.7919 |
| ASMT | acetylserotonin O-methyltransferase | 3.3093 | 0.2575 | 12.8537 |
| LINC01136 | uncharacterized LOC730227 | 3.3060 | 0.3278 | 10.0854 |
| NPAP1 | nuclear pore associated protein 1 | 3.1879 | 0.5032 | 6.3349 |
| RNU105A | RNA, U105A small nucleolar | 3.1467 | 0.3112 | 10.1118 |
| KCNRG | potassium channel regulator | 3.1419 | 0.2368 | 13.2693 |
| LINC00652 | long intergenic non-protein coding RNA 652 | 3.1288 | 0.4000 | 7.8220 |
| LOC100506023 | uncharacterized LOC100506023 | 3.1221 | 0.4824 | 6.4725 |
| IL24 | interleukin 24 | 3.1005 | 0.2146 | 14.4493 |
| NOX4 | NADPH oxidase 4 | 3.0517 | 0.4347 | 7.0194 |
| DPPA4 | developmental pluripotency associated 4 | 3.0200 | 0.4064 | 7.4304 |
| MCM3AP-AS1 | MCM3AP antisense RNA 1 | 2.9426 | 0.4110 | 7.1594 |
| FGB | fibrinogen beta chain | 2.9260 | 0.3246 | 9.0147 |
| LINC00629 | long intergenic non-protein coding RNA 629 | 2.9000 | 0.1419 | 20.4421 |
| LINC00346 | long intergenic non-protein coding RNA 346 | 2.8940 | 0.4427 | 6.5371 |
| ADORA2B | adenosine A2b receptor | 2.8421 | 0.3586 | 7.9247 |
| SNORD10 | small nucleolar RNA, C/D box 10 | 2.8343 | 0.3668 | 7.7275 |
| TP53AIP1 | tumor protein p53 regulated apoptosis inducing protein 1 | 2.8200 | 0.3417 | 8.2530 |
| LOC100507156 | uncharacterized LOC100507156 | 2.8186 | 0.3112 | 9.0575 |
| MESTIT1 | MEST intronic transcript 1, antisense RNA | 2.8129 | 0.2466 | 11.4061 |
| SRSF12 | serine/arginine-rich splicing factor 12 | 2.8033 | 0.3749 | 7.4772 |
| RNU11 | RNA, U11 small nuclear | 2.7883 | 0.3731 | 7.4744 |
| LOC100129636 | uncharacterized LOC100129636 | 2.7700 | 0.0439 | 63.1004 |
| PLGLB1 | plasminogen-like B1 | 2.7148 | 0.2139 | 12.6918 |
| CLC | Charcot-Leyden crystal protein | 2.6795 | 0.2734 | 9.8011 |
| DISC2 | disrupted in schizophrenia 2 (non-protein coding) | 2.5664 | 0.2456 | 10.4499 |
| SNORA52 | small nucleolar RNA, H/ACA box 52 | 2.5443 | 0.3149 | 8.0793 |
| C2CD4A | C2 calcium-dependent domain containing 4A | 2.5276 | 0.3776 | 6.6934 |
| SNORA24 | small nucleolar RNA, H/ACA box 24 | 2.5174 | 0.1127 | 22.3347 |
| RUNX1-IT1 | RUNX1 intronic transcript 1 (non-protein coding) | 2.4974 | 0.3603 | 6.9306 |
| SPAG6 | sperm associated antigen 6 | 2.4893 | 0.3631 | 6.8566 |
| ANXA2P2 | annexin A2 pseudogene 2 | 2.4407 | 0.3642 | 6.7009 |
| LOC100506321 | uncharacterized LOC100506321 | 2.4145 | 0.1607 | 15.0271 |
| C1orf140 | uncharacterized LOC400804 | 2.4098 | 0.2759 | 8.7332 |
| TMC1 | transmembrane channel-like 1 | 2.3852 | 0.1883 | 12.6669 |
| EEF1DP3 | eukaryotic translation elongation factor 1 delta pseudogene 3 | 2.3374 | 0.1164 | 20.0736 |
| NDST3 | N-deacetylase/N-sulfotransferase (heparan glucosaminyl) 3 | 2.2971 | 0.3751 | 6.1243 |
| SSTR5-AS1 | SSTR5 antisense RNA 1 | 2.2910 | 0.3217 | 7.1215 |
| DNM1P35 | DNM1 pseudogene 35 | 2.2767 | 0.1390 | 16.3809 |
| DENND5B-AS1 | DENND5B antisense RNA 1 | 2.2757 | 0.1697 | 13.4133 |
| LOC157273 | uncharacterized LOC157273 | 2.2729 | 0.0986 | 23.0410 |
| CLCA4 | chloride channel accessory 4 | 2.2650 | 0.0832 | 27.2169 |
| LRP4-AS1 | LRP4 antisense RNA 1 | 2.2636 | 0.3503 | 6.4611 |
| PATE2 | prostate and testis expressed 2 | 2.2521 | 0.3215 | 7.0046 |
| LOC283692 | uncharacterized LOC283692 | 2.2298 | 0.1871 | 11.9163 |
| ALG1L | ALG1, chitobiosyldiphosphodolichol beta-mannosyltransferase-like | 2.2036 | 0.2793 | 7.8890 |
| PRC1-AS1 | uncharacterized LOC100507118 | 2.1952 | 0.2936 | 7.4780 |
| HTR5AOS | uncharacterized LOC100128264 | 2.1940 | 0.0403 | 54.3903 |
| LINC00696 | long intergenic non-protein coding RNA 696 | 2.1705 | 0.1937 | 11.2037 |
| GIF | gastric intrinsic factor (vitamin B synthesis) | 2.1510 | 0.0208 | 103.1758 |
| C21orf37 | chromosome 21 open reading frame 37 | 2.0914 | 0.0456 | 45.8715 |
| LOC100128593 | uncharacterized LOC100128593 | 2.0888 | 0.1556 | 13.4248 |
| FLJ43879 | FLJ43879 protein | 2.0745 | 0.0839 | 24.7266 |
| RNF39 | ring finger protein 39 | 2.0698 | 0.3076 | 6.7282 |
| LINC01091 | uncharacterized LOC285419 | 2.0674 | 0.1969 | 10.4970 |
| RHCE | Rh blood group, CcEe antigens | 2.0550 | 0.1647 | 12.4738 |
| LOC100507053 | uncharacterized LOC100507053 | 2.0498 | 0.2998 | 6.8364 |
| CADM2-AS2 | CADM2 antisense RNA 2 | 2.0314 | 0.0512 | 39.6868 |
| CFHR1 | complement factor H-related 1 | 1.9840 | 0.1376 | 14.4161 |
| UXT-AS1 | uncharacterized LOC100133957 | 1.9581 | 0.2932 | 6.6779 |
| RNU6-35P | RNA, U6 small nuclear 35 | 1.9376 | 0.0669 | 28.9417 |
| VSX1 | visual system homeobox 1 | 1.9124 | 0.2681 | 7.1321 |
| DSCAM-AS1 | DSCAM antisense RNA 1 | 1.9012 | 0.1495 | 12.7177 |
| RBM26-AS1 | RBM26 antisense RNA 1 | 1.8998 | 0.2366 | 8.0291 |
| WDR31 | WD repeat domain 31 | 1.8724 | 0.2480 | 7.5510 |
| LOC100132078 | uncharacterized LOC100132078 | 1.8643 | 0.0439 | 42.4683 |
| GTF2IRD2P1 | GTF2I repeat domain containing 2 pseudogene 1 | 1.8517 | 0.2192 | 8.4492 |
| LOC100129083 | uncharacterized LOC100129083 | 1.8476 | 0.2871 | 6.4350 |
| ZNF221 | zinc finger protein 221 | 1.8393 | 0.2459 | 7.4788 |
| MIR205HG | MIR205 host gene (non-protein coding) | 1.7998 | 0.1205 | 14.9347 |
| SNORA40 | small nucleolar RNA, H/ACA box 40 | 1.7407 | 0.0219 | 79.6141 |
| CCDC148 | coiled-coil domain containing 148 | 1.7374 | 0.2166 | 8.0208 |
| MAP3K15 | mitogen-activated protein kinase kinase kinase 15 | 1.7336 | 0.1176 | 14.7379 |
| BLOC1S5-TXNDC5 | BLOC1S5-TXNDC5 readthrough (non-protein coding) | 1.7317 | 0.0990 | 17.4946 |
| SCARNA13 | small Cajal body-specific RNA 13 | 1.6883 | 0.0380 | 44.4695 |
| CHIAP2 | chitinase, acidic pseudogene 2 | 1.5755 | 0.0581 | 27.1000 |
| NKPD1 | NTPase, KAP family P-loop domain containing 1 | 1.5731 | 0.1668 | 9.4322 |
| CLEC19A | C-type lectin domain family 19, member A | 1.5283 | 0.2066 | 7.3972 |
| GPR78 | G protein-coupled receptor 78 | 1.5179 | 0.1951 | 7.7805 |
| PCDHB19P | protocadherin beta 19 pseudogene | 1.4955 | 0.2093 | 7.1444 |
| LINC01194 | cancer/testis antigen 49 (non-protein coding) | 1.4790 | 0.1468 | 10.0767 |
| LINC01158 | uncharacterized LOC100506421 | 1.4750 | 0.1056 | 13.9687 |
| LOC284757 | uncharacterized LOC284757 | 1.4750 | 0.1895 | 7.7840 |
| SNORA21 | small nucleolar RNA, H/ACA box 21 | 1.4693 | 0.0732 | 20.0666 |
| FAM138C | family with sequence similarity 138, member C | 1.4655 | 0.1359 | 10.7809 |
| LINC00927 | uncharacterized LOC283688 | 1.4129 | 0.0566 | 24.9577 |
| BPIFB1 | BPI fold containing family B, member 1 | 1.4105 | 0.1727 | 8.1666 |
| COL4A2-AS1 | COL4A2 antisense RNA 1 | 1.3950 | 0.0344 | 40.5443 |
| TRIM60 | tripartite motif containing 60 | 1.3826 | 0.0907 | 15.2476 |
| LOC100130417 | uncharacterized LOC100130417 | 1.3655 | 0.1702 | 8.0242 |
| CYP8B1 | cytochrome P450, family 8, subfamily B, polypeptide 1 | 1.3574 | 0.2093 | 6.4847 |
| LIPF | lipase, gastric | 1.2931 | 0.1914 | 6.7575 |
| LOC654841 | uncharacterized LOC654841 | 1.2895 | 0.1451 | 8.8881 |
| NKX6-2 | NK6 homeobox 2 | 1.2674 | 0.0605 | 20.9455 |
| DIRC1 | disrupted in renal carcinoma 1 | 1.2638 | 0.1285 | 9.8370 |
| USP50 | ubiquitin specific peptidase 50 | 1.2617 | 0.0298 | 42.2945 |
| LOC286177 | uncharacterized LOC286177 | 1.2267 | 0.1169 | 10.4889 |
| ST20-MTHFS | ST20-MTHFS readthrough | 1.2079 | 0.0088 | 137.0453 |
| LOC286359 | uncharacterized LOC286359 | 1.2074 | 0.0342 | 35.2651 |
| HTR3B | 5-hydroxytryptamine (serotonin) receptor 3B, ionotropic | 1.2026 | 0.1390 | 8.6530 |
| SNORA53 | small nucleolar RNA, H/ACA box 53 | 1.1902 | 0.0927 | 12.8380 |
| SNORA16A | small nucleolar RNA, H/ACA box 16A | 1.1840 | 0.1542 | 7.6768 |
| RHPN1-AS1 | RHPN1 antisense RNA 1 (head to head) | 1.1555 | 0.1634 | 7.0719 |
| MRAP | melanocortin 2 receptor accessory protein | 1.1479 | 0.0347 | 33.0359 |
| APOC4 | apolipoprotein C-IV | 1.1436 | 0.0085 | 134.9414 |
| CLVS1 | clavesin 1 | 1.1390 | 0.0195 | 58.4381 |
| PIH1D2 | PIH1 domain containing 2 | 1.1202 | 0.1439 | 7.7849 |
| HCG9 | HLA complex group 9 (non-protein coding) | 1.1110 | 0.1722 | 6.4514 |
| RAX2 | retina and anterior neural fold homeobox 2 | 1.0902 | 0.1288 | 8.4637 |
| LGALS17A | Charcot-Leyden crystal protein pseudogene | 1.0629 | 0.1295 | 8.2079 |
| SNORA5A | small nucleolar RNA, H/ACA box 5A | 1.0607 | 0.0502 | 21.1426 |
| MIR7-1 | microRNA 7-1 | 1.0605 | 0.1142 | 9.2831 |
| LOC388242 | coiled-coil domain containing 101 pseudogene | 1.0352 | 0.0232 | 44.5832 |
| SNORA41 | small nucleolar RNA, H/ACA box 41 | 1.0276 | 0.0871 | 11.7956 |
| LINC01016 | uncharacterized LOC100507584 | 1.0176 | 0.1092 | 9.3229 |
| CLEC4C | C-type lectin domain family 4, member C | 1.0069 | 0.0746 | 13.5017 |
| LGSN | lengsin, lens protein with glutamine synthetase domain | 1.0052 | 0.1341 | 7.4980 |

Table S7 DEGs reduced in children with SAM compared to children with stunting

| genename | description | meanfpkm_sam | meanfpkm_stunted | ratiosamstunted |
| --- | --- | --- | --- | --- |
| RNA28S5 | RNA, 28S ribosomal 5 | 7.2524 | 1104.9346 | 0.0066 |
| RNA18S5 | RNA, 18S ribosomal 5 | 6.1986 | 817.8422 | 0.0076 |
| MALAT1 | metastasis associated lung adenocarcinoma transcript 1 (non-protein coding) | 16.6771 | 792.3088 | 0.0210 |
| LOC100507412 | uncharacterized LOC100507412 | 16.2238 | 263.9371 | 0.0615 |
| RNA45S5 | RNA, 45S pre-ribosomal 5 | 0.2171 | 254.1315 | 0.0009 |
| UGT8 | UDP glycosyltransferase 8 | 9.3860 | 108.4736 | 0.0865 |
| CHRM3 | cholinergic receptor, muscarinic 3 | 4.0581 | 66.9119 | 0.0606 |
| HYPK | huntingtin interacting protein K | 1.7800 | 24.4295 | 0.0729 |
| NBPF10 | neuroblastoma breakpoint family, member 10 | 0.6893 | 13.7329 | 0.0502 |
| RPL21P28 | ribosomal protein L21 pseudogene 28 | 0.8926 | 9.7125 | 0.0919 |
| UGT1A4 | UDP glucuronosyltransferase 1 family, polypeptide A4 | 0.6362 | 8.7383 | 0.0728 |
| UGT1A5 | UDP glucuronosyltransferase 1 family, polypeptide A5 | 0.6338 | 7.0176 | 0.0903 |
| MIR941-1 | microRNA 941-1 | 0.1755 | 6.6468 | 0.0264 |
| ACTR3BP2 | ARP3 actin-related protein 3 homolog B (yeast) pseudogene 2 | 0.1805 | 4.8334 | 0.0373 |
| CECR7 | cat eye syndrome chromosome region, candidate 7 (non-protein coding) | 0.1952 | 4.2315 | 0.0461 |
| TPTE2P6 | transmembrane phosphoinositide 3-phosphatase and tensin homolog 2 pseudogene 6 | 0.1069 | 4.0831 | 0.0262 |
| LOC100289656 | Dexi homolog (mouse) pseudogene | 0.0362 | 4.0363 | 0.0090 |
| ANKRD36 | ankyrin repeat domain 36 | 0.2610 | 3.6515 | 0.0715 |
| FLNC | filamin C, gamma | 0.2533 | 3.1414 | 0.0806 |
| ACTR3BP5 | ARP3 actin-related protein 3 homolog B (yeast) pseudogene 5 | 0.0090 | 3.0944 | 0.0029 |
| RNA5S10 | RNA, 5S ribosomal 10 | 0.0355 | 3.0269 | 0.0117 |
| RNA5S2 | RNA, 5S ribosomal 2 | 0.0355 | 3.0269 | 0.0117 |
| RNA5S7 | RNA, 5S ribosomal 7 | 0.0355 | 3.0269 | 0.0117 |
| RNA5S12 | RNA, 5S ribosomal 12 | 0.0355 | 3.0269 | 0.0117 |
| RNA5S1 | RNA, 5S ribosomal 1 | 0.0355 | 3.0269 | 0.0117 |
| RNA5S17 | RNA, 5S ribosomal 17 | 0.0355 | 3.0269 | 0.0117 |
| RNA5S5 | RNA, 5S ribosomal 5 | 0.0355 | 3.0269 | 0.0117 |
| RNA5S3 | RNA, 5S ribosomal 3 | 0.0355 | 3.0269 | 0.0117 |
| RNA5S11 | RNA, 5S ribosomal 11 | 0.0355 | 3.0269 | 0.0117 |
| RNA5S15 | RNA, 5S ribosomal 15 | 0.0355 | 3.0269 | 0.0117 |
| RNA5S13 | RNA, 5S ribosomal 13 | 0.0355 | 3.0269 | 0.0117 |
| RNA5S4 | RNA, 5S ribosomal 4 | 0.0355 | 3.0269 | 0.0117 |
| RNA5S8 | RNA, 5S ribosomal 8 | 0.0355 | 3.0269 | 0.0117 |
| RNA5S6 | RNA, 5S ribosomal 6 | 0.0355 | 3.0269 | 0.0117 |
| RNA5S16 | RNA, 5S ribosomal 16 | 0.0355 | 3.0269 | 0.0117 |
| RNA5S14 | RNA, 5S ribosomal 14 | 0.0355 | 3.0269 | 0.0117 |
| NANOS2 | nanos homolog 2 (Drosophila) | 0.1574 | 2.7115 | 0.0580 |
| ZNRF2P2 | zinc and ring finger 2 pseudogene 2 | 0.1183 | 2.6876 | 0.0440 |
| FAM157B | family with sequence similarity 157, member B | 0.0193 | 2.6532 | 0.0073 |
| OR5C1 | olfactory receptor, family 5, subfamily C, member 1 | 0.0364 | 2.6229 | 0.0139 |
| ZNF843 | zinc finger protein 843 | 0.1086 | 2.2724 | 0.0478 |
| LRRTM1 | leucine rich repeat transmembrane neuronal 1 | 0.0955 | 2.0812 | 0.0459 |
| MIR941-2 | microRNA 941-2 | 0.1098 | 2.0759 | 0.0529 |
| OR1D2 | olfactory receptor, family 1, subfamily D, member 2 | 0.0155 | 2.0563 | 0.0075 |
| ANKRD30BL | ankyrin repeat domain 30B-like | 0.0424 | 1.9947 | 0.0212 |
| PENK | proenkephalin | 0.0102 | 1.8458 | 0.0055 |
| CHRFAM7A | CHRNA7 (cholinergic receptor, nicotinic, alpha 7, exons 5-10) and FAM7A (family with sequence similarity 7A, exons A-E) fusion | 0.1440 | 1.7797 | 0.0809 |
| SERF1A | small EDRK-rich factor 1A (telomeric) | 0.1562 | 1.6768 | 0.0931 |
| OR10G2 | olfactory receptor, family 10, subfamily G, member 2 | 0.0688 | 1.6017 | 0.0430 |
| AQP12B | aquaporin 12B | 0.0798 | 1.5695 | 0.0508 |
| AKR7A2P1 | aldo-keto reductase family 7, member A2 pseudogene 1 | 0.0745 | 1.5593 | 0.0478 |
| TEX38 | testis expressed 38 | 0.0621 | 1.5353 | 0.0405 |
| PLIN4 | perilipin 4 | 0.1200 | 1.5280 | 0.0785 |
| FLJ42393 | uncharacterized LOC401105 | 0.0874 | 1.5093 | 0.0579 |
| LOC401127 | WD repeat domain 5 pseudogene | 0.1117 | 1.4502 | 0.0770 |
| YY2 | YY2 transcription factor | 0.1148 | 1.4344 | 0.0800 |
| PCDHA4 | protocadherin alpha 4 | 0.0036 | 1.3292 | 0.0027 |
| OR4Q3 | olfactory receptor, family 4, subfamily Q, member 3 | 0.0062 | 1.3253 | 0.0047 |
| VPS13A-AS1 | VPS13A antisense RNA 1 | 0.0517 | 1.3231 | 0.0391 |
| C16orf3 | chromosome 16 open reading frame 3 | 0.1169 | 1.3190 | 0.0886 |
| KRTAP3-3 | keratin associated protein 3-3 | 0.0267 | 1.2919 | 0.0206 |
| YBX3P1 | Y box binding protein 3 pseudogene 1 | 0.0033 | 1.2856 | 0.0026 |
| OR4N3P | olfactory receptor, family 4, subfamily N, member 3 pseudogene | 0.0760 | 1.2776 | 0.0594 |
| VWC2 | von Willebrand factor C domain containing 2 | 0.0817 | 1.2661 | 0.0645 |
| LOC100133050 | glucuronidase, beta pseudogene | 0.0781 | 1.2631 | 0.0618 |
| POM121L4P | POM121 transmembrane nucleoporin-like 4 pseudogene | 0.0298 | 1.2622 | 0.0236 |
| OR2W3 | olfactory receptor, family 2, subfamily W, member 3 | 0.0333 | 1.2481 | 0.0267 |
| HRASLS | HRAS-like suppressor | 0.0424 | 1.1798 | 0.0359 |
| OR1F2P | olfactory receptor, family 1, subfamily F, member 2 | 0.1033 | 1.1778 | 0.0877 |
| ODF4 | outer dense fiber of sperm tails 4 | 0.0967 | 1.1729 | 0.0824 |
| RFPL4A | ret finger protein-like 4A | 0.0736 | 1.1510 | 0.0639 |
| CARTPT | CART prepropeptide | 0.0495 | 1.1476 | 0.0432 |
| KRTAP5-3 | keratin associated protein 5-3 | 0.0607 | 1.1083 | 0.0548 |
| NEFM | neurofilament, medium polypeptide | 0.0898 | 1.1081 | 0.0810 |
| SUMO4 | SMT3 suppressor of mif two 3 homolog 4 (S. cerevisiae) | 0.0410 | 1.0886 | 0.0376 |
| NYAP1 | neuronal tyrosine-phosphorylated phosphoinositide-3-kinase adaptor 1 | 0.0817 | 1.0588 | 0.0771 |
| HIST2H2BA | histone cluster 2, H2ba (pseudogene) | 0.0724 | 1.0447 | 0.0693 |
| C11orf42 | chromosome 11 open reading frame 42 | 0.0779 | 1.0327 | 0.0754 |
| GALR3 | galanin receptor 3 | 0.0555 | 1.0220 | 0.0543 |
| CLK2P | CDC-like kinase 2, pseudogene | 0.0498 | 1.0115 | 0.0492 |

**Figure S1** Ingenuity Pathway Analysis (IPA) of differentially regulated genes using pairwise comparisons between groups. Group-wise comparisons were assessed for (A) canonical pathway enrichment, (B) enrichment of upstream regulators, and (C) associated diseases and functions.
